# Dapagliflozin ameliorates diabetes-induced spermatogenic dysfunction by modulating the adenosine metabolism along the gut microbiota-testis axis

**DOI:** 10.1038/s41598-024-51224-2

**Published:** 2024-01-05

**Authors:** Zirun Jin, Yalei Cao, Qi Wen, Haitao Zhang, Zhuofan Fang, Qiancheng Zhao, Yu Xi, Zhichao Luo, Hui Jiang, Zhe Zhang, Jing Hang

**Affiliations:** 1 Department of Urology, Center for Reproductive Medicine, Peking University Third Hospital, No. 49 North Garden Road, Haidian District, Beijing, 100191 China; 2 Department of Urology, Department of Andrology, Peking University First Hospital, No. 8, Xishiku Street, Xicheng District, Beijing, 100034 China; 3 Department of Obstetrics and Gynecology, Center for Reproductive Medicine, State Key Laboratory of Female Fertility Promotion, Peking University Third Hospital, No.49 North Garden Road, Haidian District, Beijing, 100191 China; 4 Key Laboratory of Assisted Reproduction, Ministry of Education, Beijing, China; 5 Beijing Key Laboratory of Reproductive Endocrinology and Assisted Reproduction, Beijing, China; 6 National Clinical Research Center for Obstetrics and Gynecology, Beijing, China

**Keywords:** Infertility, Type 2 diabetes, Microbiome

## Abstract

Male infertility is one of the most common complications of diabetes mellitus (DM). Dapagliflozin is widely used to manage the type II DM. This study aimed to assess the dapagliflozin’s effects on the spermatogenesis by administering either dapagliflozin (Dapa) or vehicle (db) to male *db/db* mice, and using littermate male *db/m* mice as the control (Con). We further performed the integrative analyses of the cecal shotgun metagenomics, cecal/plasmatic/testicular metabolomics, and testicular proteomics. We found that dapagliflozin treatment significantly alleviated the diabetes-induced spermatogenic dysfunction by improving sperm quality, including the sperm concentration and sperm motility. The overall microbial composition was reshaped in Dapa mice and 13 species (such as *Lachnospiraceae bacterium* 3–1) were regarded as potential beneficial bacteria. Metabolites exhibited modified profiles, in which adenosine, cAMP, and 2′-deoxyinosine being notably altered in the cecum, plasma, and testis, respectively. Testicular protein expression patterns were similar between the Dapa and Con mice. In vivo results indicated that when compared with db group, dapagliflozin treatment alleviated apoptosis and oxidative stress in testis tissues by down-regulating 2′-deoxyinosine. This was further validated by in vitro experiments using GC-2 cells. Our findings support the potential use of dapagliflozin to prevent the diabetes-induced impaired sperm quality and to treat diabetic male infertility.

## Introduction

Infertility affects approximately 8%–12% of couples of reproductive-ages, with nearly half of these cases attributed to male factors^1^. The complicated mechanisms underlying male infertility, including congenital genetic defects, oxidative stress, environmental factors, and metabolic abnormalities such as endocrine disruption and diabetes, can all result in dysfunctional spermatogenesis and impaired sperm maturation^2–10^. These sophisticated pathogenic mechanisms have resulted in satisfactory treatment outcomes for male infertility^11^.

Diabetes mellitus (DM), especially type II diabetes mellitus (T2DM), is becoming increasingly prevalent, even among younger individuals^12^. Male infertility is a common complication of DM^9,13^. Several anti-diabetic drugs, such as insulin and metformin, have shown protective effects on male reproduction by multiple approaches like restoring the function of hypothalamic-pituitary-gonad axis and diminishing the testicular apoptosis^14–17^. On the contrary, hypoglycemic drug glibenclamide has been found to decrease sperm viability in ejaculated human semen samples^18^. Dapagliflozin, which blocks sodium glucose cotransporter type 2 (SGLT2) in the renal proximal tubules, thereby facilitating glucosuria, reducing plasma glucose levels and thus improved glycemic parameters, has emerged as a novel, safe, and effective clinical treatment for DM by exerting multiple metabolic benefits such as reducing the risk of cardiovascular events and attenuating diabetic kidney diseases^19–22^. We also found that dapagliflozin could alleviate diabetes-induced spermatogenic dysfunction by activating the GLP-1R/phosphatidylinositol 3-kinase (PI3K)/Akt signaling pathway and inhibiting the apoptosis in the testis tissues of diabetic mice^23^.

The gut microbiota (GM), also known as the intestinal microbiota, plays a crucial role in maintaining overall body homeostasis and has a significant impact on spermatogenesis^24^. Disturbance of GM composition is closely associated with spermatogenic impairment, as GM dysbiosis-induced polyamine metabolism disruption can result in testicular dysfunction^25–27^. Meanwhile, the therapeutic effect of dapagliflozin on diabetes is also achieved by reducing the ratio of *Firmicutes* to *Bacteroidetes* and improving the abundance of *Akkermansia muciniphila* in GM of type II diabetic mice^28–30^. However, whether dapagliflozin has reproductive effects and the underlying mechanisms remain largely unclear.

In the present study, we discovered a protective effect of dapagliflozin on recovering the spermatogenic dysfunction of *db/db* diabetic mice. We examined the GM compositions and profiled the cecal/plasmatic/testicular metabolic as well as testicular proteomic patterns. Moreover, we performed in vivo and in vitro studies to explore the potential protective mechanisms of dapagliflozin on spermatogenic dysfunction in *db/db* diabetic mice. Our results provide novel insights into the protective effects of dapagliflozin and its potential as a therapeutic treatment for diabetic male infertility.

## Materials and methods

### Ethics statement

All experiments were performed in accordance with the ethical policies and procedures approved by the Animal Care and Use Committee of Peking University (approval number: LA2021371).

### Animal model

Six-week-old male BKS.Cg-Dock7^m^+/+Lepr^db^/Nju (*db/db*) mice (Nanjing Biomedical Research Institution of Nanjing University, Nanjing, China) were group-housed conventionally and maintained on a 12-h light/dark cycle at 23.0 ± 3.0 °C with free access to food and water. The ARRIVE Essential 10 guideline was used to formulate the study design, sample preparation, results observation, and data analysis.

After two weeks of adaptation, the diabetic condition of the mice was confirmed by the fasting blood glucose level ≥ 11.1 mmol/L or the random blood glucose level ≥ 16.7 mmol/L. The diabetic mice were randomly assigned into two groups and treated with either dapagliflozin (labeled as Dapa) or the vehicle (ddH_2_O, labeled as db) (n = 8 for each group). The mice were intragastrically administered 1 mg/kg dapagliflozin (gifted by AstraZeneca Pharmaceutical Co Ltd., London, UK) or vehicle daily for five weeks. The dose of dapagliflozin has been described in a previous study^31^. Eight littermate *db/m* mice of the same age were used as normal controls (labeled as Con). The mice were anesthetized with sodium pentobarbital (50 mg/kg, intraperitoneally) and euthanized with a sodium pentobarbital overdose in 6 mg/ml solution^32,33^.

### Body weight and blood glucose measurement

The random body weight of the mice was measured from 8:00 am to 9:30 am, and the fasting body weight was measured after 15 h of overnight fasting. All blood samples were collected from the tail vein, and blood glucose was measured by the glucose oxidase method using a hand-held OneTouch Ultra glucometer (LifeScan, Milpitas, CA, USA). If the glucose level was higher than 33.3 mmol/L (upper detection limit of the glucometer), the value was recorded as 33.3 mmol/L.

### Hematoxylin and eosin (H&E) staining

Under deep anesthesia, the mice testis were removed quickly and fixed in 10% neutral buffered formalin for 24 h. Following dehydration through an ethanol series, the fixed testes were embedded in paraffin and then sectioned. Paraffin sections (5-µm-thick) were then stained with H&E as previously described^34^. Histological analysis was performed using a light microscope (Leica DM 4000, Germany).

### Sperm count and motility assessment

Cauda epididymal sperm of the mice were collected and prepared as described previously^35^. In brief, two caudal epididymis samples were placed into human tubal fluid medium containing 97.8 mM NaCl, 4.69 mM KCl, 4 mM NaHCO_3_, 0.37 mM KH_2_PO_4_, 2.04 mM CaCl_2_, 0.2 mM MgCl_2_, 21.4 mM lactic acid, 21 mM HEPES, 2.78 mM glucose, and 0.33 mM Na-pyruvate as well as penicillin (100 IU/mL) and streptomycin (100 μg/mL), adjusted to pH 7.4 with NaOH. Then, the cauda epididymis was slightly cut into three pieces and incubated in a 5% CO_2_ incubator at 37 °C for 5 min. The sperm were gently filtered through nylon gauze, centrifuged, and then suspended in 1 mL fresh M199 medium (Gibco). Semen analysis was performed by a computer-assisted semen analysis (CASA) system (WLJY-9000, Beijing Weili New Century Science and Technology Development Co., Ltd, China) according to the laboratory manual of the World Health Organization for sperm concentration and sperm motility^36^. The following parameters were evaluated: rapid progressive motility (grade A, %), progressive motility (grade A + B, %), straight-line velocity (VSL, μm/s), curve-line velocity (VCL, μm/s), average path velocity (VAP, μm/s), linearity (LIN, %), amplitude of lateral head displacement (ALH, μm), and straightness (STR, %). The sperm concentration, expressed as × 10^6^/mL, was determined by the hemocytometer method on two separate preparations of the semen sample. A minimum of 200 sperm were counted for each assay.

### Immunofluorescence staining

Testis tissues were fixed in 4% paraformaldehyde, embedded in paraffin, and then cut into 5 µm sections. The paraffin sections were heated in an autoclave in citrate buffer and preincubated in permeabilization blocking buffer (0.1 mmol/L PBS, pH 7.3, 0.5% (wt/vol) Triton X-100). The sections were blocked for 60 min with 10% (vol/vol) donkey serum. The testicular sections were stained with primary antibodies at 4 °C overnight and secondary antibodies for one hour at room temperature, followed by washing and staining with DAPI (Beyotime, Jiangsu, China) for 10 min at room temperature; then, the sections were observed under a confocal microscope (Zeiss LSM710, Carl Zeiss Microscopy GmbH, Jena, Germany) at excitation wavelengths of 488 nm (green) and 405 nm (blue). The primary antibodies were as follows: rabbit polyclonal anti-DAZL (1:100; ab34139, Abcam, Cambridge, UK); mouse monoclonal anti-SYCP3 (1:100; ab97672, Abcam); rabbit polyclonal anti-TNP1 (1:300; ab73135, Abcam); rabbit polyclonal anti-PGK2 (1:300; D121803, Sangon Biotechnology, Shanghai, China) and rabbit monoclonal anti-WT1 (1:50; ab89901, Abcam). The secondary antibodies were as follows: Cy3-conjugated AffiniPure donkey polyclonal anti-mouse IgG (H + L) and Alexa Fluor 488-conjugated AffiniPure donkey polyclonal anti-rabbit IgG (H + L) (both 1:500; Jackson ImmunoResearch Laboratories, Philadelphia, PA, USA).

### Shotgun metagenomic sequencing and analysis

Genomic DNA of cecal digesta samples was extracted using a QIAGEN kit and monitored by electrophoresis on a 1% agarose gel. The quality of the DNA samples was further quantified using a Qubit^®^ 2.0 fluorometer (Life Technologies, CA, USA) with an OD value between 1.8 and 2.0. For library construction, a total of 1 μg of DNA per sample was used as input material for the DNA sample preparations. Sequencing libraries were generated using the NEBNext^®^ UltraTM DNA Library Prep Kit for Illumina (NEB, USA) following the manufacturer’s recommendations, and index codes were added to attribute sequences to each sample. Briefly, the DNA sample was fragmented by sonication to a size of 350 bp, and then DNA fragments were end-polished, A-tailed, and ligated with the full-length adaptor for Illumina sequencing with further PCR amplification. Finally, PCR products were purified (AMPure XP system), and libraries were analyzed for size distribution by using an Agilent2100 Bioanalyzer and quantified using real-time PCR. After the index-coded sample clusters were generated on a cBot Cluster Generation System according to the manufacturer’s instructions, the library preparations were sequenced on an Illumina NovaSeq platform, and paired-end reads were generated, with at least 6 Gb reads per sample.

We used Readfq (V8, https://github.com/cjfields/readfq) to acquire the clean data for subsequent analysis. Host sequences were then discarded by mapping the sequences against the reference genome (hg19) using BowTie2.2.4 (http://bowtie-bio.sourceforge.net/bowtie2/index.shtml). We pooled and subjected the remaining set of clean reads to metagenomics by using SOAPdenovo software (V2.04, http://soap.genomics.org.cn/soapdenovo.html). Then, we interrupted the assembled scaftigs from the N connection and left the scaftigs without N. All samples' clean data were compared to each scaffold by using Bowtie2.2.4 software to acquire the PE reads that were not used. The assembled scaftigs (> 500 bp) were predicted as ORFs by MetaGeneMark (V2.10, http://topaz.gatech.edu/GeneMark/) software, and the length information shorter than 100 nt was filtered. CD-HIT software (V4.5.8, http://www.bioinformatics.org/cd-hit) was adopted for redundancy and to obtain the unique initial gene catalog and ORF prediction. DIAMOND software (V0.9.9, https://github.com/bbuchfink/diamond/) was used to BLAST the unigenes to the sequences of bacteria, fungi, archaea, and viruses, which were all extracted from the NR database (Version: 2018-01-02, https://www.ncbi.nlm.nih.gov/). We used the LCA algorithm, which is applied to the system classification of MEGAN software, to ensure the species annotation information of sequences. We adopted DIAMOND software (V0.9.9) to BLAST unigenes to the functional database. The functional database includes the KEGG database (Version 2018-01-01, http://www.kegg.jp/kegg/)^37–39^, eggNOG database (Version 4.5, http://eggnogdb.embl.de/#/app/home), and CAZy database (Version 201,801, http://www.cazy.org/). For each sequence's BLAST result, the best BLAST hit was used for subsequent analysis.

### Non-targeted metabolic profiling

Gut digesta, plasma, and testis samples were subjected to metabonomic analysis separately. Small intestine digesta samples were collected, immediately frozen in liquid nitrogen and stored at − 80 °C. The samples were removed and thawed on ice. We homogenized the samples with 20% acetonitrile and 80% methanol for plasma, and 70% methanol for gut digesta and testis for 20 s and then centrifuged (3000 rpm, 4 °C) for 30 s. Then, 400 μL of 70% methanol water internal standard extractant was added, the sample was shaken (1500 rpm) for 5 min and placed on ice for 15 min. The sample was then centrifuged (12,000 rpm, 4 °C) for 10 min, and 300 μL of the supernatant was transferred to − 20 °C for 30 min. Finally, we centrifuged (12,000 rpm, 4 °C) the sample for 3 min and used the supernatant for analysis. All samples were acquired by the LC–MS system following machine orders. The analytical conditions were as follows: UPLC: column, Waters ACQUITY UPLC HSS T3 C18 (1.8 µm, 2.1 mm*100 mm); column temperature, 40 °C; flow rate, 0.4 mL/min; injection volume, 2 μL; solvent system, water (0.1% formic acid): acetonitrile (0.1% formic acid); gradient program, 95:5 V/V at 0 min, 10:90 V/V at 11.0 min, 10:90 V/V at 12.0 min, 95:5 V/V at 12.1 min, and 95:5 V/V at 14.0 min.

Similarly, blood samples were mixed with heparin sodium and centrifuged (3000 rpm, 4 °C) for 10 min to obtain the supernatant plasma, and testicular samples were pretreated as with small intestine digesta samples. The following metabolomics procedures were the same as those used for gut digesta.

Unsupervised principal component analysis (PCA) was performed by using the statistics function PR comp within R (www.r-project.org). The data were unit variance scaled before unsupervised PCA. The hierarchical cluster analysis results of the samples and metabolites were presented as heatmaps with dendrograms, while Pearson correlation coefficients between samples were calculated by the cor function in R and presented as only heatmaps. Significantly regulated metabolites between groups were determined by VIP ≥ 1 and absolute Log2FC (fold change) ≥ 1 and *p* value < 0.05. VIP values were extracted from the OPLS-DA results, which also contained score plots and permutation plots, and were generated using the R package MetaboAnalystR. The data were log transformed (log2) and mean centered before OPLS-DA. To avoid overfitting, a permutation test (200 permutations) was performed. Identified metabolites were annotated using the KEGG compound database (http://www.kegg.jp/kegg/compound/), and annotated metabolites were then mapped to the KEGG pathway database (http://www.kegg.jp/kegg/pathway.html). Significantly enriched pathways were identified with a hypergeometric test *p* value for a given list of metabolites.

### Label-free proteomic analysis

We weighed and ground testicular samples into cell powder using a mortar and liquid nitrogen. Then, we added four volumes of lysis buffer (8 M urea, 100 mM NaCl, 100 mM Tris–HCl, pH 8.0, 1% protease inhibitor, 1% phosphatase inhibitor) and treated the cells with sonication. The cell debris was removed by centrifugation at 12,000 g at 4 °C for 10 min, and the supernatant was collected. The total protein concentration was determined with a BCA kit (Thermo Fisher Scientific, USA) following the manufacturer’s instructions. The protein quantity and volume from each sample were adjusted to the same value, followed by the addition of TCA to a final concentration of 20% and the precipitation of proteins at 4 °C for 2 h. The protein pellets were collected by centrifugation at 4500 × g for 5 min and washed with precooled acetone 3 times. Then, 100 mM TEAB was added, and the precipitate was dispersed by ultrasound. We used trypsin at a 1:50 mass ratio for the overnight digestion. Then, the solutions were reduced with 5 mM dithiothreitol for 30 min at 56 °C and alkylated with 11 mM iodoacetamide (IAA) for 15 min at room temperature in darkness. The digested peptides were desalted with C18 SPE (3M) according to the manufacturer’s instructions.

The tryptic peptides were dissolved in solvent A (0.1% formic acid in 2% acetonitrile) and then separated on a homemade reversed-phase analytical column (25-cm length, 100 μm i.d.) using a NanoElute Ultra-Performance Liquid Chromatography (UPLC) system. The gradient comprised an increase from 7 to 24% over 72 min and from 24 to 32% over 12 min, an increase to 80% in 3 min, and a hold at 80% for the last 3 min. A constant flow rate of 450 nL/min was maintained. The peptides separated by the UPLC system were then subjected to ionization by both electron spray (the Capillary) and captive spray source, followed by tandem mass spectrometry (MS/MS) in time of flight (TOF) Pro (Bruker Daltonics, USA) for analysis. The electrospray voltage applied was 1.6 kV. The peptide parent ions and their secondary fragments were detected and analyzed in TOF. The m/z scan range was 100 to 1700 for the MS2 spectrum. The data acquisition used a parallel accumulation serial fragmentation (PASEF) procedure. We selected precursors with charge states of 0 to 5 for fragmentation, and 10 PASEF-MS/MS scans were acquired per cycle; we set the dynamic exclusion time as 30 s to avoid repeated scanning of parent ions. The elution gradient was set as follows: 0–50 min, 2~22%; 50–52 min, 22~35%; 52–55 min, 35~90%; 55–60 min, 90%. The flow rate was constant at 300 nL/min. The resulting MS/MS data were processed using the Maxquant search engine (v.1.6.6.0). Tandem mass spectra were searched against the human SwissProt database concatenated with the reverse decoy database. Trypsin was specified as a cleavage enzyme allowing up to 2 missing cleavages. The minimum length of the peptide segment was set as 7 amino acid residues, and the maximum modification number of the peptide segment was set to 5. The mass tolerance for precursor ions was set as 20 ppm in the first search and 20 ppm in the main search, and the mass tolerance for fragment ions was set as 0.02 Da. Alkylation (cysteine) was specified as a fixed modification, while oxidation (methionine), acetylation (protein N-term), and desamidization (asparagine and glutamine) were specified as variable modifications. The false discovery rates (FDRs) of both protein identification and peptide spectrum matching (PSM) were set to 1%. We compared the expression of each protein with a paired *t-*test and determined differentially expressed proteins (DEPs) with a |fold change (FC)|≥ 2.0 and *p* value < 0.05. We used statistical analysis methods, including PCA and Pearson’s correlation coefficient, to test sample repeatability. Statistical significance (*p* < 0.05) was assessed by using Student’s *t-*test. For the annotation and enrichment of DEPs, the Kyoto Encyclopedia of Genes and Genomes (KEGG) database was used to classify all identified proteins by a 2-sided Fisher’s exact probability test. The KEGG with an adjusted *p* value < 0.05 was considered significant. To find the functional correlation of different groups between DEPs, we performed clustering analysis by hierarchical clustering and visualized it by a heatmap based on *p* values from Fisher’s exact test. The horizontal dimension of the heatmap represented Fisher’s exact test results of different groups, while the longitudinal dimension described functional classification.

### Cell culture and treatment

The GC-2 spd cell line (CL-0593, Procell Life Science & Technology Co., Ltd, Wuhan, China) was cultured in DMEM supplemented with 10% fetal bovine serum (FBS, Hyclone), 1% penicillin and streptomycin at 37 °C in a humidified atmosphere containing 5% CO_2_. To mimic diabetic inflammatory stimuli in vitro, GC-2 cells in all group were treated with palmitic acid (PA, 100 μM) for 48h. In Dapa group, GC-2 cells were treated with Dapa (30 μM) for 48h. In 2′-Deo group, GC-2 cells were treated with Dapa (30 μM) for 24 h, then 2′-deoxyinosine (100 μM) were added into the medium for another 24 h^40^.

### Western blot analysis

A piece of testis tissues or GC-2 cells were homogenized in ice-cold RIPA lysis buffer (Beyotime) containing 1 mM phenylmethanesulfonyl fluoride (PMSF). The homogenates were centrifuged at 12,000 g for 10 min at 4 °C to yield the total protein extract in the supernatant, and then analyzed by Western blotting. The concentration of protein was measured with a bicinchoninic acid (BCA) assay kit (Thermo Scientific), and an equal amount of protein samples (30 μg for testis tissues and 25 μg for GC-2 cell lines) was denatured and then separated through SDS-PAGE using 10% separating gels and transferred to a PVDF membrane (Bio-Rad, Hercules, CA). The membranes were blocked with 5% nonfat milk in TBST buffer for 60 min at room temperature and then incubated with the following primary antibodies at 4 °C overnight: rabbit polyclonal anti-XIAP (1:1000, 10,037-1-1g, Proteintech); rabbit monoclonal anti-Caspase-3 (1:1000, 9662S, Cell Signaling Technology, CST); rabbit monoclonal anti- Caspase-8 (1:1000, 4790S, CST); mouse monoclonal anti-Caspase-9 (1:1000, 9508S, CST), rabbit monoclonal anti-BCL2 (BCL2, 1:1000, Ab182858, Abcam), rabbit polyclonal anti-Bax (1:1000, 14796S, CST), mouse monoclonal anti-α-tubulin (1:3000, 3873S, CST), respectively. The blots were incubated in horseradish peroxidase-conjugated secondary antibody including goat anti-rabbit or mouse IgG antibody (1:5000, Biodragon Immunotechnologies, Suzhou, Jiangsu, China). Protein bands were visualized using an enhanced chemiluminescence detection kit followed by using a Tanon 5200 chemiluminescence detection system (Tanon, Shanghai, China). The bands were quantified with a computer-assisted imaging analysis system (Image J, NIH).

### Oxidative stress assessments

We used commercial kits purchased from Beyotime Biotechnology according to the manufacturer’s instructions to assess the oxidative stress in testis tissues of mice as described before^41^. Total antioxidant capacity (cat# S0119), superoxide dismutase (SOD) (cat# S0109, total superoxide dismutase assay kit with NBT), and glutathione peroxidase (GPx) (cat# S0058, total glutathione peroxidase assay kit with NADPH) activities as well as the levels of malondialdehyde (MDA) (cat# S0131M, lipid peroxidation MDA assay kit) and hydrogen peroxide (cat# S0038, hydrogen peroxide assay kit) were measured.

### Flow cytometric analysis

To analyze the effects of the indicated treatments on cell survival, cells were detected by Annexin V-FITC and PI Detection Kit (C1062M, Beyotime) following the manufacturer’s protocol. Flow cytometry data were assessed using BD FACSDiva Software v7.0 (Becton–Dickinson, USA). The intracellular ROS levels were detected using a peroxide-sensitive fluorescent probe (DCFH-DA; Beyotime) according to the instructions of the manufacturer. Briefly, DCFH-DA was diluted to a final concentration of 10 μM for 30 min at 37 °C, then the cells were harvested and washed twice with PBS and subjected to flow cytometry.

### Statistical analysis

Statistical analyses were performed with GraphPad Prism 8.0 for Windows (GraphPad Software, La Jolla, CA). All quantitative biochemical data and immunofluorescence staining were representative of at least three independent experiments. One-way ANOVA with Sidak's post hoc or two-tailed unpaired Student's *t* test was used for multiple comparisons or the mean values between two groups. All data are expressed as the mean ± SEM, and differences with *p* < 0.05 were considered statistically significant.

## Results

**Figure 1:**
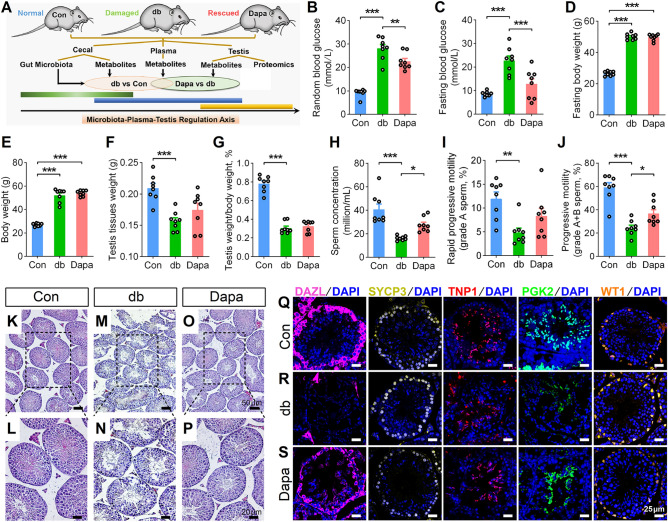
Effects of dapagliflozin on spermatogenetic dysfunction in *db/db* diabetic mice. (**A**) The experimental pipeline of metagenomic, metabolomic and proteomic analysis. (**B–C**) The random (**B**) and fasting (**C**) blood glucose levels of *db/m* (control, Con), *db/db* (db) and dapagliflozin-treated *db/db* (Dapa) mice. (**D–E**) The fasting (**D**) and random (**E**) body weights of mice. (**F–G**) The testis tissue weight (**F**) and testis index (ratio of testis weight and body weight) (**G**) of mice. (**H–J**) Sperm concentration (**H**) and sperm motility, including grade A sperm (**I**) and grade A + B sperm (**J**). (**K–M**) Hematoxylin and eosin (H&E) staining of *db/m* (**K**), *db/db* (**L**) and dapagliflozin-treated *db/db* mice (**M**). Scale bar = 50 μm (**K**, **M**, **O**) or 20 μm (**L**, **N**, **P**). (**Q–S**) Representative images of immunofluorescence staining for DAZL (marker of spermatogonia), SYCP3 (marker of spermatocytes), TNP1 (marker of spermatids), PGK2 (marker of spermatozoa) and WT1 (marker of Sertoli cell). Scale bar = 25 μm. All data are presented as mean ± SEM. *P < 0.05; **P < 0.01; ***P < 0.001. One-way ANOVA with Sidak's *post-hoc* test. n = 8 mice per group.

As indicated in the experimental pipeline (Fig. 1A), we collected the cecum digesta samples, plasma samples, and testis tissues from three groups: normal *db/m* mice (control group, Con), damaged *db/db* mice (diabetic group, db), and dapagliflozin-treated *db/db* mice (Dapa). These samples were subjected to shotgun metagenomics, metabolomics, or proteomics, respectively. We employed multiple comparison groups including db_vs_Con (comparing db to Con) and Dapa_vs_db (comparing Dapa to db) and applied an intersectional strategy to identify the effects of dapagliflozin. This strategy considered that the overlapping parts between these two comparisons, which involved ones that were upregulated in db_vs_Con but downregulated in Dapa_vs_db, as well as the ones that were downregulated in db_vs_Con but upregulated in Dapa_vs_db, to be rescued by dapagliflozin.

### Dapagliflozin attenuates the anthropometric and spermatogenetic aberrance of db/db mice

We measured the blood glucose levels and body weights in three groups and found a deterioration of these parameters in db mice compared to Con (Fig. 1B–G). However, dapagliflozin treatment resulted in decreased random and fasting blood glucose levels (Fig. 1B–C), yet there was no significant difference in fasting and random body weights (Fig. 1D–E; Fig. S1A), nor the testis tissue weight and testis index (Fig. 1F–G; Fig. S1B). Using computer-assisted sperm analysis (CASA), we observed a reduction in sperm concentration and sperm motility, including grade A sperm and grade A + B sperm, in *db/db* mice (Fig. 1H–J). Importantly, dapagliflozin treatment significantly rescued these impairments, leading to increased sperm concentration and progressive motility (Fig. 1H,J). Other parameters of sperm motility, including straight-line velocity, curve-line velocity and average path velocity, were consistently decreased in db mice but not altered in Dapa (Fig. S1C–H).

Next, we performed histological examinations to evaluate the spermatogenesis in three groups. We discovered a reduction in the number of spermatogenic cells and spermatids in the seminiferous tubules and disorganization of the seminiferous tubules in testis tissues in the db group (Fig. 1K–N), while these phenomena were greatly alleviated in the Dapa group (Fig. 1O,P). Consistently, immunofluorescence staining demonstrated that protein expression levels of DAZL (the marker of spermatogonia), SYCP3 (the marker of spermatocytes), TNP1 (the marker of spermatids), and PGK2 (the marker of spermatozoa) were reduced in the testis tissues of db group but ameliorated by dapagliflozin treatment (Fig. 1Q–S). These findings indicate the ameliorative effect of dapagliflozin on the diabetes-induced spermatogenesis. Overall, our results suggest that dapagliflozin administration may attenuate the pathological damage of spermatogenesis in *db/db* diabetic mice.

### Dapagliflozin ameliorates GM dysbiosis in db/db mice

**Figure 2:**
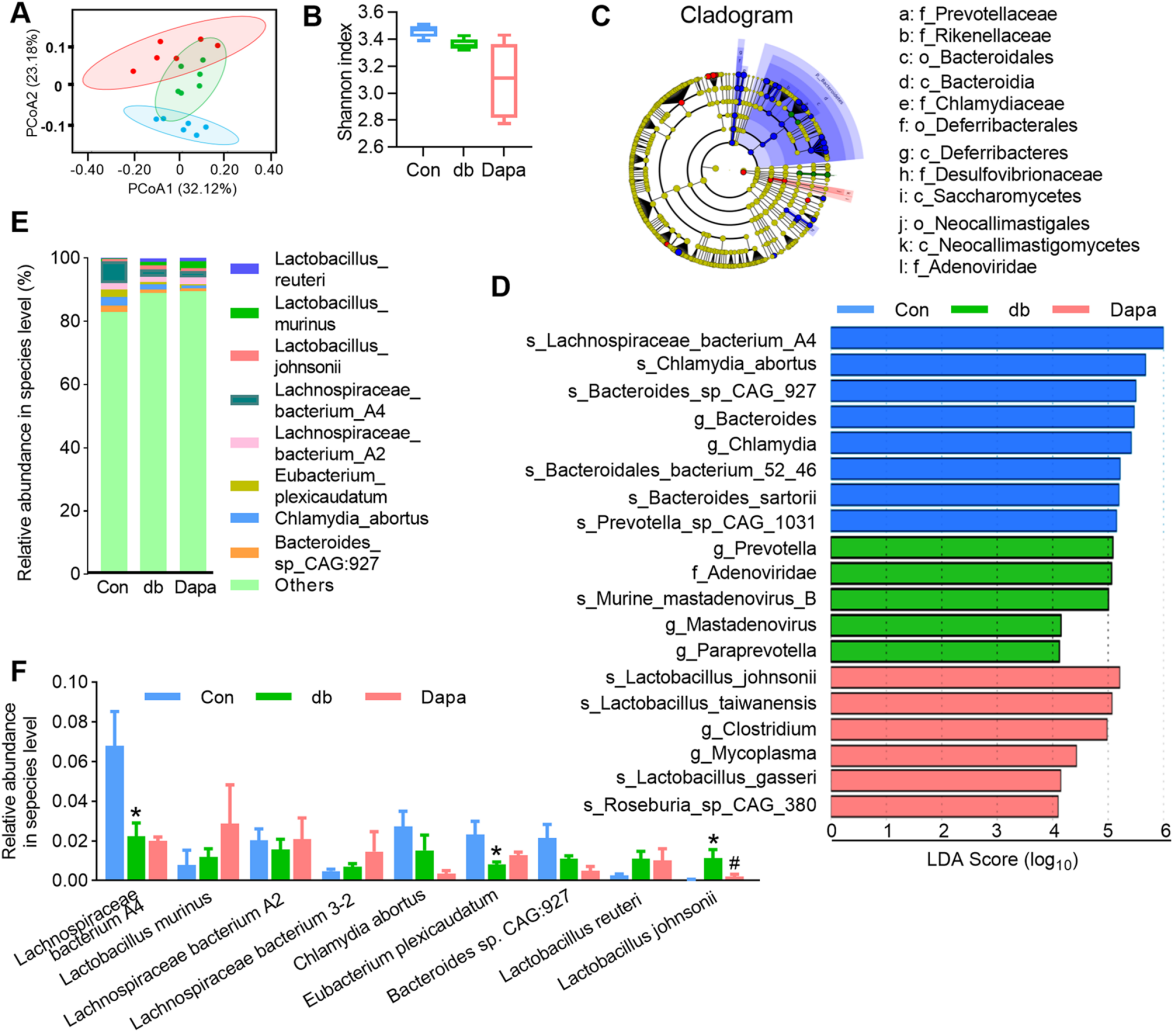
Dapagliflozin treatment altered the taxonomic composition of microbial communities. (**A**) Principal coordinate analysis (PCoA) plots based on Bray–Curtis distances. (**B**) Shannon index to describe the alpha diversity of gut bacterial assemblages in the mice. (**C–D**) Statistical differences at the species level between the Con, db, and Dapa groups were identified using the line discriminant analysis (LDA) effect size (LEfSe) method. Cladogram illustrating the output of the LEfSe algorithm. Significantly distinct taxonomic nodes are colored, and the branch areas are shaded according to the effect size of the taxa (**C**). Taxa enriched in the Con (Blue), db (Green), and Dapa (Red) groups are indicated by LDA scores (**D**). (**E**) Histogram of the top nine species. (**F**) Changes in abundance at the species level. All data are presented as mean ± SEM. *P < 0.05 for db; #*P *< 0.05 for Dapa. One-way ANOVA with Sidak's *post-hoc* test. n = 5–6 mice per group.

To comprehensively evaluate the effects of dapagliflozin treatment on *db/db* mice, we performed shotgun metagenomic sequencing of cecal digesta samples to investigate how dapagliflozin influences the GM composition and distribution. Principal coordinate analysis (PCoA) based on Bray–Curtis distances (β-diversity) revealed that microbial composition was significantly different among the three groups (Fig. 2A). Meanwhile, the α-diversity, assessed by the Shannon index, exhibited no significant differences across the groups (Fig. 2B). To identify the key phylotypes that were significantly altered in Dapa, we employed the linear discriminant analysis (LDA) effect size (LEfSe) method to validate and analyze the sequences. At the genus level, *Prevotella* and *Paraprevotella* were enriched in the db group, whereas at the species level, *Lactobacillus johnsonii* (*L. johnsonii*)*, Lactobacillus taiwanensis* (*L. taiwanensis*)*, Lactobacillus gasseri,* and *Roseburia-sp-CAG-380* were enriched in the Dapa group (Fig. 2C–D). These differences indicated that dapagliflozin reshaped the microbial community structure of the GM in *db/db* mice.

Furthermore, we compared the overall GM composition by analyzing the degree of bacterial taxonomic similarity at the phylum, genus, and species levels. *Firmicutes* and *Bacteroidetes* were the two dominant phyla across the groups (Fig. S2A), and the abundances of certain phyla were reduced while others were increased after dapagliflozin treatment (Fig. S2B). At the genus level, the abundances of *Bacteroides*, *Chlamydia*, *Prevotella*, *Eubacterium,* and *Roseburia* were changed among the three groups (Fig. S2C–D). In addition, our high-quality metagenomics data allowed us to analyze the GM composition at the species level, especially identifying the nine most abundant species (Fig. 2E–F). Using the intersectional strategy mentioned earlier, we identified 13 species that were significantly counteracted by dapagliflozin treatment, including *Oscillibacter sp.1–3*, *Lachnospiraceae bacterium 3–1*, *Helicobacter rodentium, Lachnospiraceae bacterium A2* and *Lachnospiraceae bacterium M18-1* (Fig. S3, highlighted in red). Interestingly, only one species, *L. johnsonii*, showed not only high abundance but also significant difference among the three groups (Fig. 2F and Fig. S3), indicating a potential crucial role for this specific bacterium. These results indicate the GM dysbiosis in the *db/db* diabetic mice may partially be ameliorated by dapagliflozin.

### Dapagliflozin treatment counteracts alterations in metabolites in db mice

**Figure 3:**
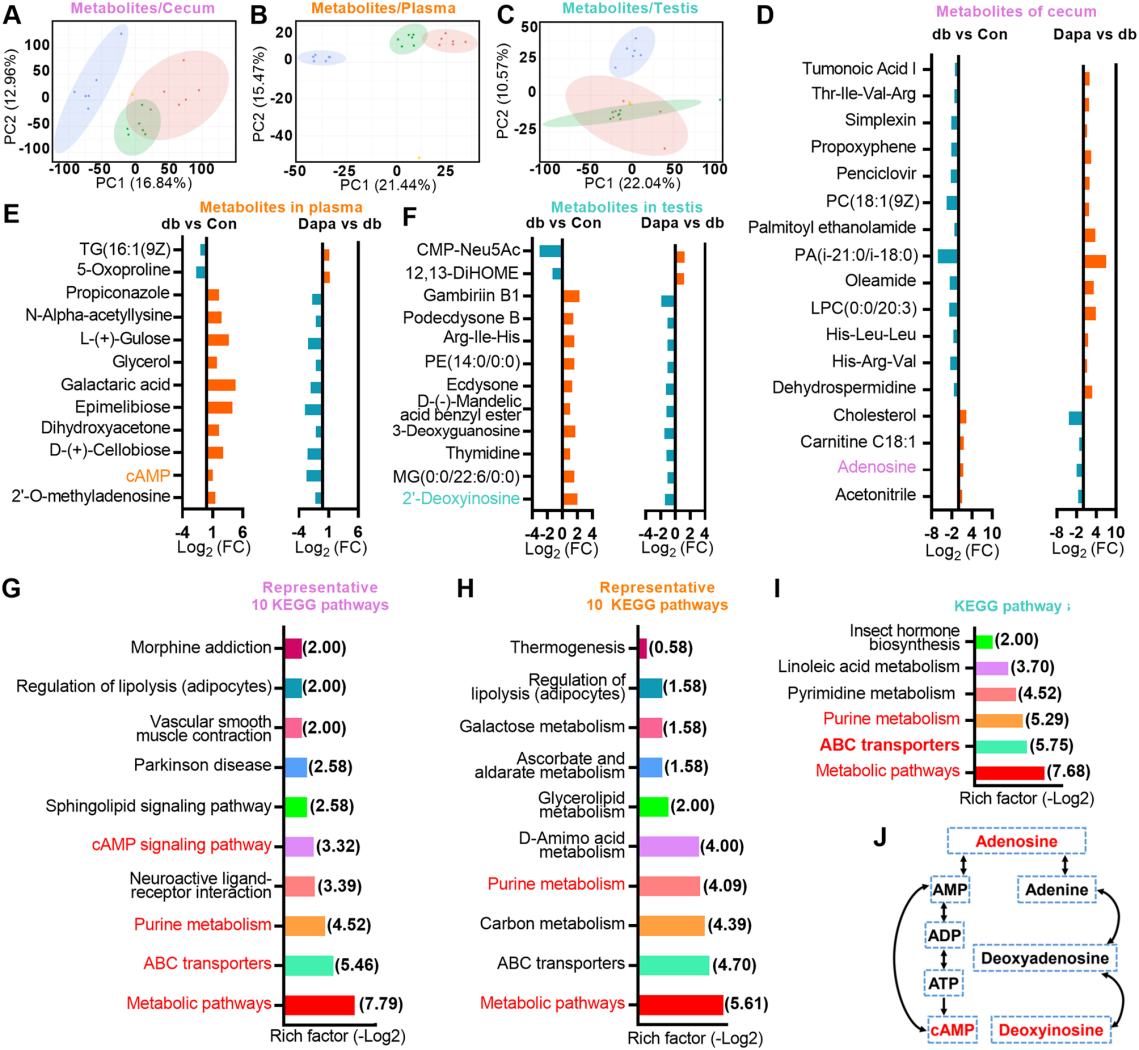
Alterations in microbial, plasmatic, and testicular metabolic profiles caused by dapagliflozin administration. (**A–C**) Principal component analysis (PCA) plots of metabolomic profiles from cecum (**A**), plasma (**B**), and testis (**C**). (**D–F**) The Log2-fold change (Log_2_FC) of the 17, 12 and 12 metabolites that were rescued by dapagliflozin in cecum (**D**), plasma (**E**) and testis samples (**F**). (**G–I**) Representative 10, 10 and 6 KEGG pathways of the dapagliflozin rescued microbial (**G**), plasmatic (**H**) and testicular metabolites (**I**). Rich factor was shown as -Log2 and the enriched KEGG pathways involved adenosine, cAMP and deoxyinosine were presented in red. (**J**) The relationship of adenosine, cAMP and deoxyinosine in the purine metabolism pathway.

We then conducted non-targeted metabolome profiling of the intestine, plasma, and testis samples for further exploration (Supplementary Data S1). PCA revealed distinct patterns of dominant metabolites among the groups (Fig. 3A–C). Initially, we applied k-means clustering to illustrate the overall abundance tendencies for all metabolites. We observed there was comparative tendency of alterations in subclass 2 in the cecum, subclass 4 in the plasma and subclass 6 in the testis, which exhibited low level in Con, high level in db and low level in Dapa, suggesting detrimental effects were rescued by dapagliflozin (Fig. S4). The intensity of some representative metabolites was shown in Fig. S5. Subsequently, using the aforementioned intersectional strategy, we found that 17 microbiotic metabolites in intestine were rescued in Dapa group, as demonstrated by their log2-fold change (Log_2_FC) illustrating 13 beneficial metabolites versus four detrimental ones (Fig. 3D). Similarly, 12 rescued metabolites (two beneficial and ten detrimental) in the plasma and 12 rescued metabolites (two beneficial and ten detrimental) in the testis were also identified (Fig. 3E–F).

To gain deeper insights into the underlying regulatory network, we performed the KEGG pathway enrichment analysis and identified several enriched pathways for these metabolites in cecum, plasma and testis, respectively (Fig. 3G–I). Two pathways, purine metabolism and metabolic pathways, exhibited intriguing overlap. Further investigation of the metabolites involved in these pathways identified three associated metabolites that could be converted to each other: adenosine in cecum, adenosine 3′,5′-cyclic monophosphate (cAMP) in plasma, and 2′-deoxyinosine in testis (Fig. 3J and Fig. S5). Altogether, these results implicate that dapagliflozin administration could influence the intestinal, plasmatic, and testicular metabolism in the *db/db* diabetic mice, and the aberrant regulation of the cAMP metabolic pathway may be a major contributor to these alterations.

### Dapagliflozin treatment alleviates apoptosis and oxidative stress in the testis tissues of db/db mice

**Figure 4:**
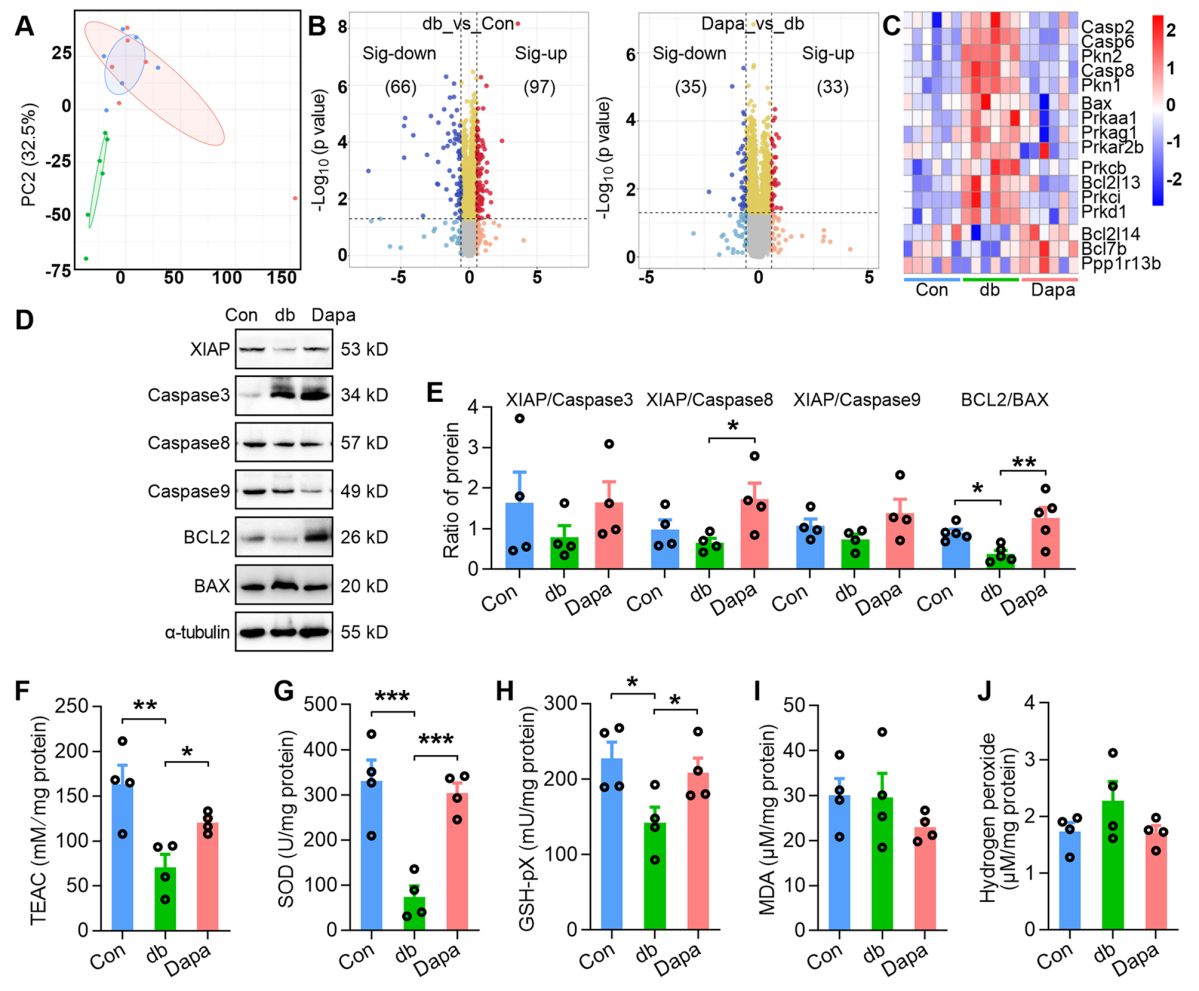
Dapagliflozin treatment alleviates apoptosis and oxidative stress in the testis tissues of *db/db* mice. (**A**) PCA plots of testis tissue proteomic profiles. (**B**) The volcano plots of the altered proteins between the db and Con groups (db_vs_Con) or between the Dapa and db groups (Dapa_vs_db). (**C**) The heat map of proteins associated with apoptosis and cAMP signaling pathway from proteomic profiles. Red indicates high abundance and blue indicates low abundance. (**D–E**) Protein expression of XIAP, Caspase 3, Caspase 8, Caspase 9, BCL2 and BAX (**D**) and ratio of protein (**E**) in three groups. n = 4–5 mice per group. The original images of all gels have been cropped, and the above original images can be seen in the Supplementary File (4D). (**F–J**) Oxidative stress related indicators including total antioxidant capacity (**F**), total GPx (**G)**, SOD activities (**H**), malondialdehyde (MDA) (**I**), and hydrogen peroxide (**J**). For (**E**–**J**), P values were determined by One-way ANOVA with *Sidak's* post-hoc test, *P < 0.05; **P < 0.01; ***P < 0.001, compared with vehicle or Dapa group.

To further assess the potential protective mechanisms of dapagliflozin on spermatogenic dysfunction in *db/db* diabetic mice, we performed a label-free LC–MS/MS proteomic analysis on testis tissues of all three groups. PCA showed that the Dapa and Con groups principally overlapped but were distinct from db (Fig. 4A), indicating a similarity in protein expression patterns between the Dapa and Con groups. Volcano plots in Fig. 4B showed that 66/35 and 97/33 proteins were down- and up-regulated in db_vs_Con and Dapa_vs_db, respectively. Molecular function (MF) analysis of these regulated proteins showed that many MF entries, such as hormone binding and signaling receptor binding, were upregulated in db_vs_Con and downregulated in Dapa_vs_db (Fig. S6). As previously mentioned (Fig. 1K–S), dapagliflozin rescued the decrease in spermatogenic cells of *db/db* mice, indicating that dapagliflozin may regulate the apoptosis pathway. Indeed, dapagliflozin widely regulated proteins participated in apoptosis, including Casp2/6/8, BAX, BCL2L13, BCL2L14, and BCL7b, as well as proteins related to cAMP signaling pathway, such as PKN1, PRKAA1, PRKAG1 and PKD1 (Fig. 4C). We conducted anti-apoptotic protein/pro-apoptotic protein ratio to show the expression patterns of apoptotic-related genes in testis tissues^42^. Consistently, the ratio of XIAP/Caspase8 and BCL2/BAX, quantified by Western blot, was increased in Dapa group compared with db (Fig. 4D–E, Fig. S7). Furthermore, we investigated whether oxidative stress was induced or inhibited by assessing several oxidative stress-related indicators. We found that total antioxidant capacity (Fig. 4F), SOD activities (Fig. 4G), and total GPx expression (Fig. 4H), was augmented in the testis tissues of db mice but alleviated by dapagliflozin treatment, while malondialdehyde (MDA) and hydrogen peroxide levels remained unchanged (Fig. 4I,J). We also observed the changes in some molecules associated with oxidative stress and redox levels: the intensity of cysteine and glutathione were changed in db mice, and γ-L-Glutamate-Cysteine was increased in Dapa mice (Fig. S8).

Furthermore, we performed an integrative analysis using multi-omics factor analysis (MOFA) to help generate statistically proven trans-omics relationships. We identified three factors with a minimum explained variance of 2% in at least one data type (Fig. S9A). All three factors were active only in testis proteome assays (Fig. S9B). They major accounted for 25.3% of the variance in proteomic data (Fig. S9C). Absolute loadings of the top features of factors 1–3 in the proteomic data and heatmap of the top 20 proteins of factor 2 from proteome array were shown in Fig. S8D–G. Collectively, these results suggest that the dapagliflozin treatment can partially rescue apoptosis and oxidative stress in the testis tissues of diabetic mice.

### 2′-deoxyinosine treatment reverses the alleviation effects of dapagliflozin on apoptosis involving oxidative stress

**Figure 5:**
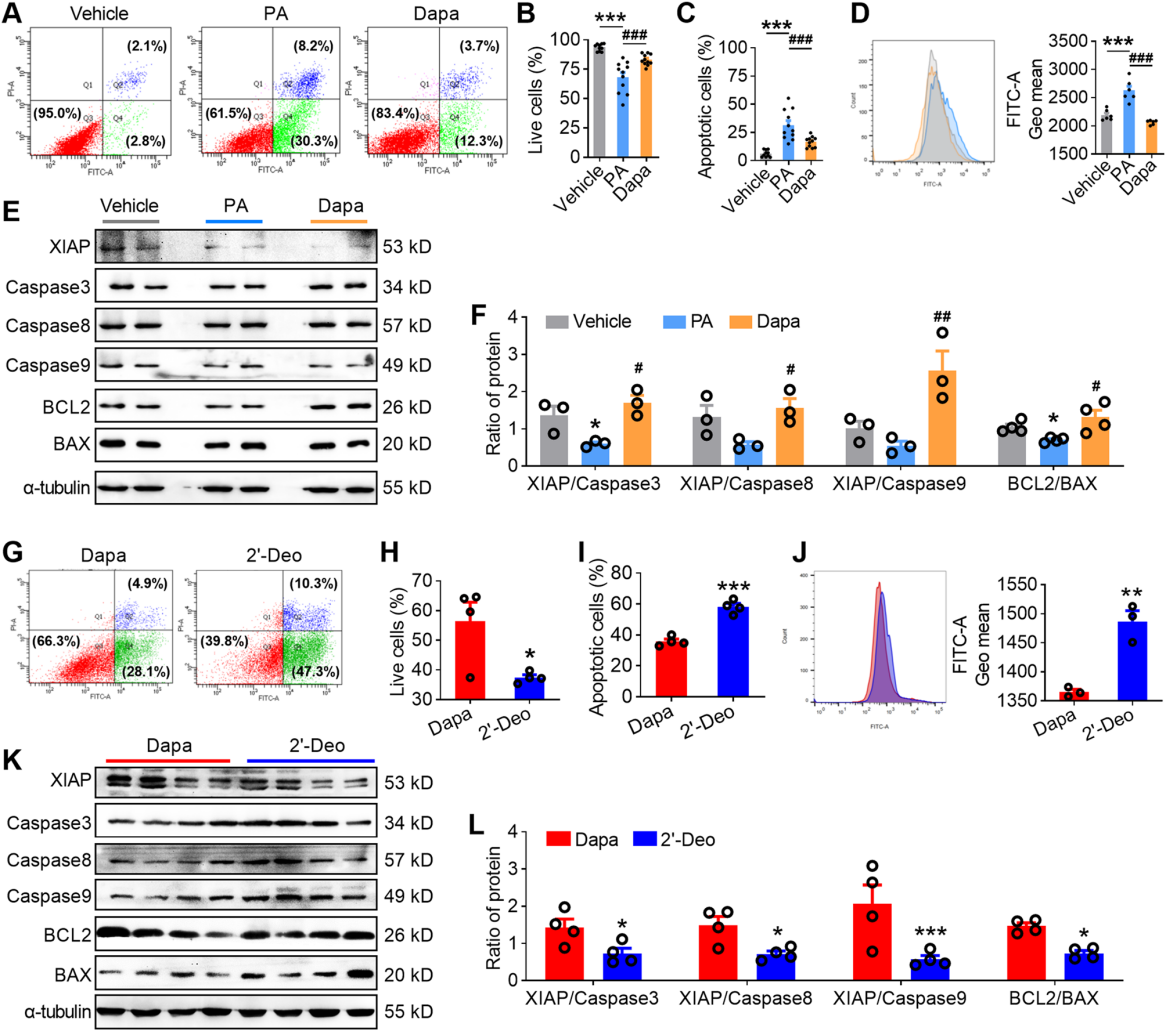
2′-deoxyinosine treatment reversed alleviation effects of Dapa on apoptosis and oxidative stress in GC-2 cells. (**A–C**) Typical scatter plots (**A**) and summary (**B**–**C**) of Annexin V-FITC/PI assay of GC-2 cells in vehicle, PA and Dapa groups. GC-2 cells in PA and Dapa groups were treatment with 100 μM palmitic acid (PA) or 100 μM palmitic acid (PA) mixed with 30 μM Dapa. 48 h later, cells were harvested for subsequent experiments. n = 11 per group. (**D**) ROS level of GC-2 cells in three groups. Left: representative image; right: statistics of the ROS level. n = 6 per group. (**E–F**) Protein expression of XIAP, Caspase 3, Caspase 8, Caspase 9, BCL2 and BAX (**E**) and ratio of protein (**F**) in three groups. n = 3–4 per group. (**G–I**) Typical scatter plots (**G**) and summary (**H**–**I**) of Annexin V-FITC/PI assay of GC-2 cells in Dapa and 2′-deoxyinosine groups. GC-2 cells were treatment with 100 μM palmitic acid (PA) mixed with 30 μM Dapa for 24 h, then 0.1% DMSO or 100 μM 2′-deoxyinosine was added into the medium. 24 h later, cells were harvested for subsequent experiments. n = 3 per group. (**J**) ROS level of GC-2 cells in three groups. Left: representative image; right: statistics of the ROS level. (**K–L**) Protein expression of XIAP, Caspase 3, Caspase 8, Caspase 9, BCL2 and BAX (**K**) and ratio of protein (**L**) in three groups. n = 4 per group. All data are presented as mean ± SEM. For **B**, **C**, **D** and **F**, P values were determined by One-way ANOVA with *Sidak's* post-hoc test, *, #, *P *< 0.05; **, ##, *P *< 0.01; ***, ^###^, *P *< 0.001, compared with vehicle or Dapa group. For **H**, **I**, **J** and** L**, P values were determined by two-tailed unpaired Student's *t* test. *P < 0.05; **P < 0.01; ***P < 0.001. The original images of all gels in this Figure have been cropped, and the above original images can be seen in the Supplementary File (5E, 5K).

**Figure 6:**
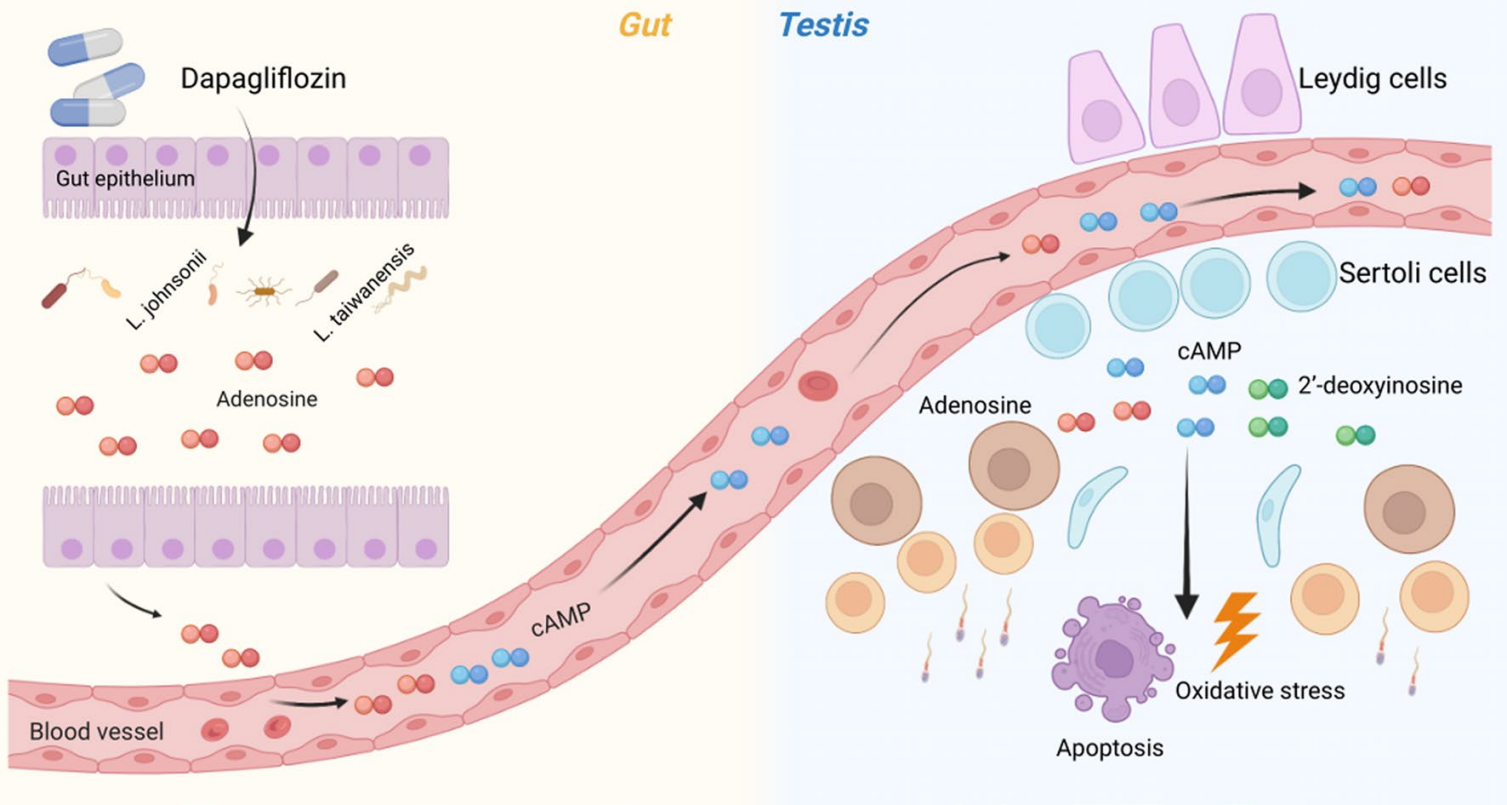
Diagram for the possible mechanisms of the protective effects of dapagliflozin on GM-plasma-testis tissue axis. For db mice, the altered GM abundances in intestine, especially *L. johnsonii*, were closely related to the changed cecal adenosine metabolism; subsequently, the metabolized cAMP entered into circulation, resulting in the aberrant plasmatic cAMP enrichment and testicular 2′-deoxyinosine. Dapagliflozin administration could alleviate apoptosis and oxidative stress in the testis tissues by down-regulating the intensity of 2′-deoxyinosine, thus improved spermatogenesis of *db/db* diabetic mice. Taken together, we suggest that the protective effect of dapagliflozin on spermatogenetic aberrance is associated with the GM-plasma-testis regulatory axis.

As we observed that dapagliflozin treatment counteracted the increased intensity of 2′-deoxyinosine in testis of *db/db* mice (Fig. 3F), we performed in vitro studies to evaluate whether 2′-deoxyinosine treatment could reverse the therapeutic effects of dapagliflozin on palmitic acid (PA)-treated GC-2 cells. We first established the diabetic GC-2 spd cells by treating them with palmitic acid (PA) (Fig. S10). Flow cytometry data showed that dapagliflozin treatment alleviated the apoptotic rate (Fig. 5A–C) and the production of ROS (Fig. 5D) of PA-treated GC-2 cells. Moreover, dapagliflozin treatment significantly increased the ratio of XIAP/Caspase3, XIAP/Caspase8, XIAP/Caspase9, and BCL2/BAX of PA-treated GC-2 cells (Fig. 5E–F, Fig. S11). These therapeutic effects of dapagliflozin treatment on PA-treated GC-2 cells could be reversed by 2′-deoxyinosine treatment, as evidenced by increased levels of apoptosis and ROS (Fig. 5G–J) as well as a decreased ratio of XIAP/Caspase3, XIAP/Caspase8, XIAP/Caspase9 and BCL2/BAX (Fig. 5K–L). Taken together, our results highlight that the protective effect of dapagliflozin on spermatogenetic aberrance is associated with the rescued apoptosis and oxidative stress along a GM-plasma-testis regulatory axis (Fig. 6).

## Discussion

Diabetes-induced spermatogenetic disorder has become a global public health problem. Dapagliflozin is widely used for the treatment of diabetes and the related complications, yet its impact on male infertility remains poorly understood. In the present study, we found the dapagliflozin treatment would ameliorate the apoptosis and oxidative stress, improve the sperm quality, and protect the spermatogenesis of *db/db* diabetic mice.

Previous research has demonstrated that several antidiabetic drugs can protect male reproduction through various mechanism^14–17^, and GM dysbiosis is known to contribute to male reproductive dysfunction by influencing the process of spermatogenesis and the permeability of the blood-testis barrier^25–27,43^. Modulating the GM through drug treatment or fecal microbiota transplantation (FMT) has been shown to ameliorate these effects^44–46^. High-fat diet (HFD) decreases male fertility via upsetting GM and transplantation of alginate oligosaccharide improves GM to alleviate HFD-induced male infertility^47^. For instance, metformin alters the upper small intestinal microbiota and benefits T2DM treatment^48,49^. However, the effects of dapagliflozin on the fecal microbiome are still controversial. While some researchers found *Proteobacteria* are enriched after dapagliflozin treatment^29^, others reported no significant impact on GM α-diversity or composition in T2DM^28^. Our studies revealed that the overall microbial composition at the phylum level was similar between the Con and db groups. A very interesting phenomenon was that although *Lactobacillus* was the most abundant genus and remained generally unchanged in the three groups, one species, *L. johnsonii*, was dramatically increased in the db mice but almost recovered in the Dapa mice. On the contrary, some other researchers reported that *L. johnsonii* MH-68 supplementation mitigates the development of diabetes by attenuating glycemic levels^50,51^. We speculate that despite belonging to the same genus, different species may possess different functions and the interactions between species would affect the molecular function of the microbiome.

The metabolites can benefit male reproduction through the GM-plasma-testis axis: dietary intervention with amino acids modulates the GM composition and improves reproductive function^52,53^, and alginate oligosaccharides rescue busulfan-disrupted spermatogenesis by upregulating the circulation levels of glutathione and gamma-glutamylcysteine^46,54^. The microbial alterations can influence the host metabolism and hormone, SOD, GPx levels in blood plasma, which may serve as a non-invasive biomarker to predict the testicular injury^55–57^. Our results showed that dapagliflozin treatment counteracted the altered levels of adenosine of intestine, cAMP of plasma, and 2′-deoxyinosine of testis in *db/db* mice. The cAMP signaling pathway has important roles in regulating spermatogenesis^58,59^, with excessive cAMP concentrations leading to male infertility^60^. The restoration of adenosine, cAMP, and 2′-deoxyinosine levels indicates a potential cascade regulation of cAMP signaling in dapagliflozin’s regulation on spermatogenesis and oxidative stress. Notably, cAMP is involved in many molecular functions especially in oxidative stress and cell apoptosis by cAMP-PKA signaling pathway^61,62^. Accordingly, dapagliflozin treatment also altered the protein expression patterns such as cAMP signaling pathway and apoptosis-associated protein of *db/db* mice. Dapagliflozin may improve spermatogenesis in diabetic mice by reducing adenosine in cecum, cAMP in plasma and 2′-deoxyinosine in testis. We also found that accumulation of 2′-deoxyinosine induced ROS production and cell apoptosis and weakened the ameliorative effect of dapagliflozin in PA-treated GC-2 cells. Taken together, dapagliflozin could ameliorate diabetes-induced spermatogenic dysfunction by targeting the adenosine metabolism through gut microbiota-testis axis.

Nevertheless, some limitations exist in our study: whether the effect of dapagliflozin on spermatogenic conditions depends on its hypoglycemic effect is unclear, and further research should be carried out to verify the causal relationship between specific intestinal microorganisms and metabolites and confirm the mechanism of metabolites and protein expression using either animal models or in vitro experiments.

## Conclusions

Taken together, our results demonstrate that dapagliflozin may protect against diabetic spermatogenesis dysfunction through the attenuated GM dysbiosis, restored metabolites, modulated apoptosis, and alleviated oxidative stress status via the GM-plasma-testis tissue axis, thus providing a novel mechanism for dapagliflozin treatment in diabetic male infertility.

### Supplementary Information

Supplementary Figures.

Supplementary Figures.

Supplementary Data S1.

## Data Availability

The raw metagenomic sequencing reads generated during this study are freely available at SRA (https://www.ncbi.nlm.nih.gov/sra/) under accession number PRJNA847564 (https://www.ncbi.nlm.nih.gov/bioproject/PRJNA847564). Non-targeted metabolome of the gut, plasma, and testis samples of mice are presented in Supplementary Data S1. The mass spectrometry proteomics data have been deposited to the ProteomeXchange Consortium via the PRIDE partner repository with the dataset identifier PXD034622.

## Abbreviations

ALH: Amplitude of lateral head displacement
ANOVA: Analysis of Variance
cAMP: Adenosine 3′, 5′-cyclic monophosphates
CASA: Computer-assisted semen analysis
DAPI: 4′,6-Diamidino-2-phenylindole
DAZL: Deleted in azoospermia-like
DEPs: Differentially expressed proteins
eggNOG: Evolutionary genealogy of genes: Non-supervised Orthologous
FMT: Fecal microbiota transplantation
GM: Gut microbiota
GO: Gene ontology
H&E: Hematoxylin and eosin
IAA: Iodoacetamide
KEGG: Kyoto Encyclopedia of Genes and Genomes
LC–MS/MS: Liquid Chromatography–Mass Spectrometry and Liquid Chromatography–Tandem Mass Spectrometry
LDA: Linear discriminant analysis
LEfSe: Linear discriminant analysis (LDA) effect size
LIN: Linearity
Log_2_FC: Log2 fold change
MS: Mass Spectrometer
ORF: Open Reading Frame
PASEF: Parallel accumulation serial fragmentation
PBS: Phosphate-buffered saline
PCA: Principal component analysis
PCoA: Principal-coordinate analysis
PGK2: Phosphoglycerate kinase 2
PKA: Protein Kinase A
PKC: Protein Kinase C
PKD1: Protein kinase D1
SEM: Standard error of mean
SGLT2: Sodium-glucose co-transporter type 2
STR: Straightness
SYCP3: Synaptonemal complex protein 3
T2DM: Type II diabetes mellitus
VAP: Average path velocity
VCL: Curve-line velocity
VSL: Straight-line velocity
WT1: Wilms tumor protein homolog
