# Supplementary material for: Dapagliflozin ameliorates diabetes-induced spermatogenic dysfunction by modulating the adenosine metabolism along the gut microbiota-testis axis
Source: Sci Rep. 2024 Jan 5;14:641. doi: 10.1038/s41598-024-51224-2 (PMC10770392; doi:10.1038/s41598-024-51224-2)
Supplement: Supplementary file 1 — Supplementary Figures. [file 41598_2024_51224_MOESM1_ESM.pdf]

Figure 4D

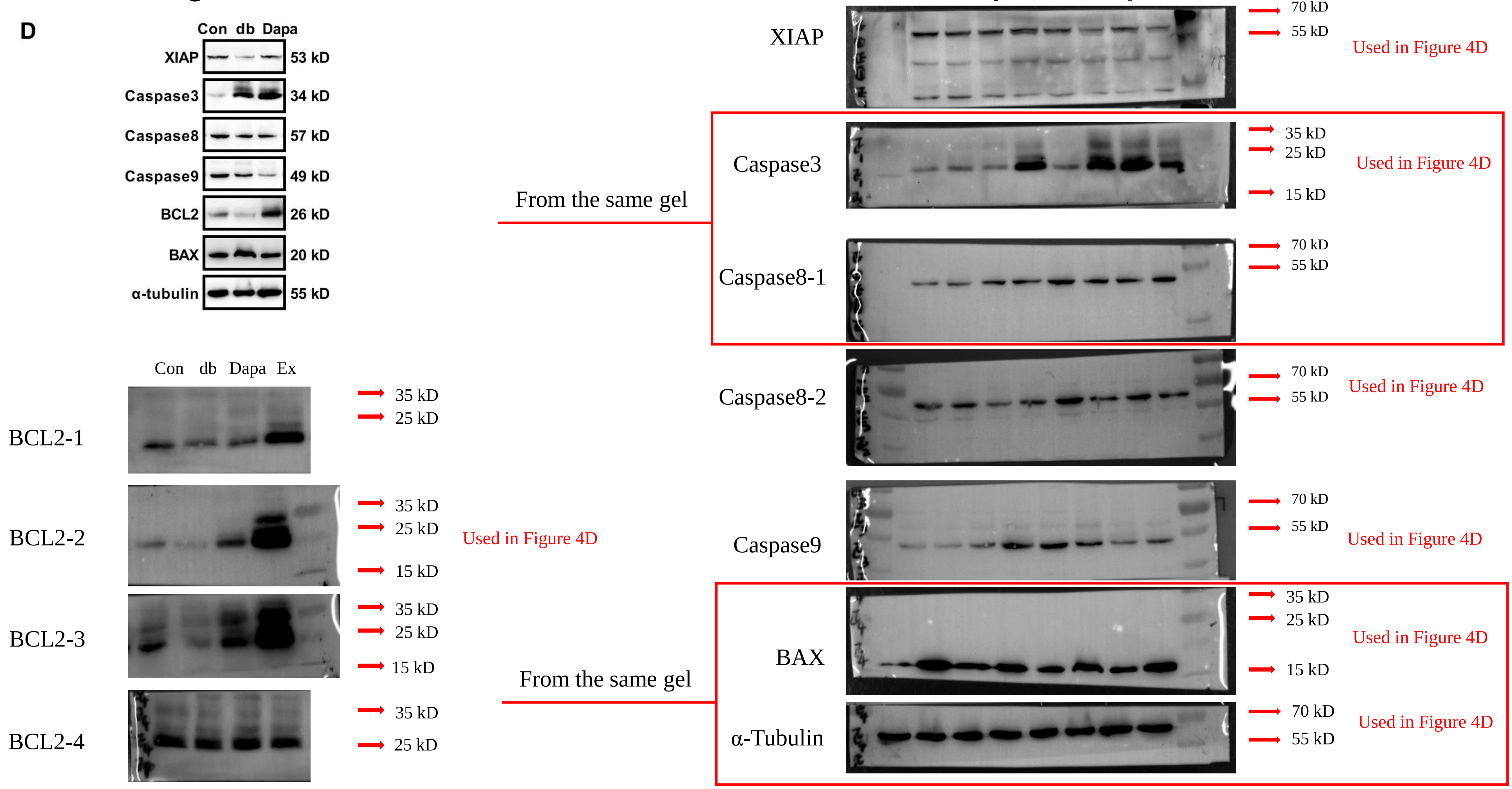
D
Con db Dapa
XIAP 53 kD
Caspase3 34 kD
Caspase8 57 kD
Caspase9 49 kD
BCL2 26 kD
BAX 20 kD
α-tubulin 55 kD
Con db Dapa Ex
BCL2-1
35 kD
25 kD
BCL2-2
35 kD
25 kD
15 kD
Used in Figure 4D
BCL2-3
35 kD
25 kD
15 kD
BCL2-4
35 kD
25 kD
Con db Dapa Ex Con db Dapa Ex
XIAP
70 kD
55 kD
Used in Figure 4D
From the same gel
Caspase3
35 kD
25 kD
15 kD
Used in Figure 4D
Caspase8-1
70 kD
55 kD
Caspase8-2
70 kD
55 kD
Used in Figure 4D
Caspase9
70 kD
55 kD
Used in Figure 4D
From the same gel
BAX
35 kD
25 kD
15 kD
Used in Figure 4D
α-Tubulin
70 kD
55 kD
Used in Figure 4D

Figure 5E

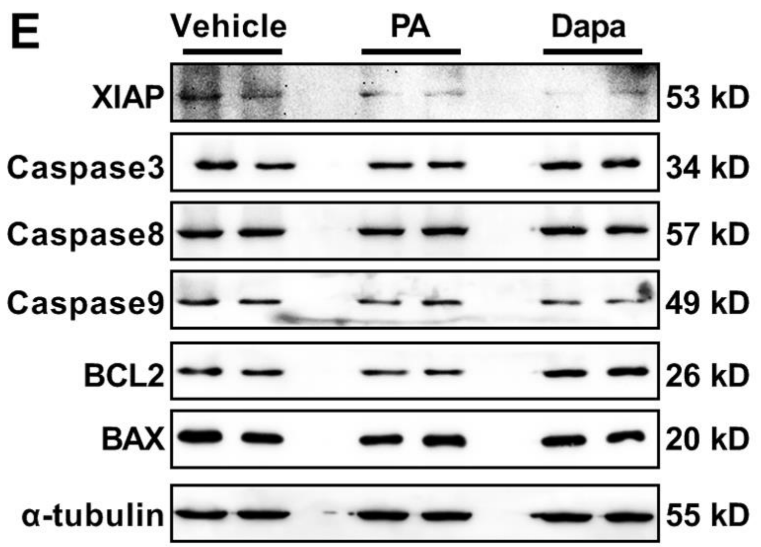

From the same gel

From the same gel

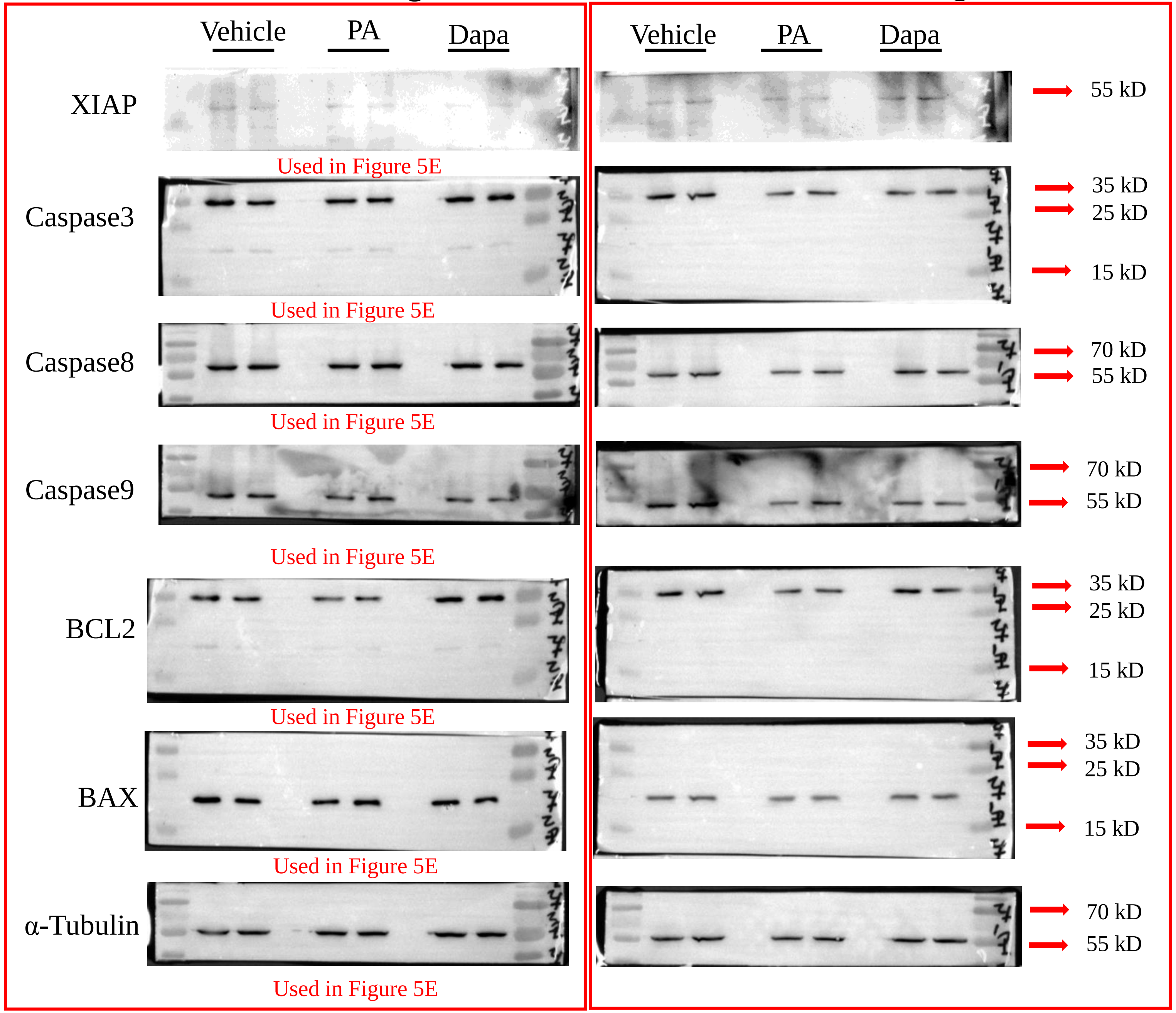

Figure 5K

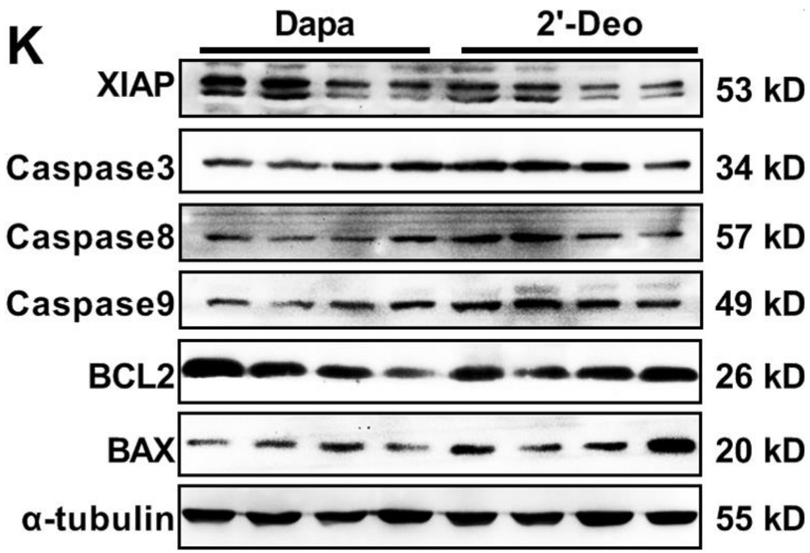
K
Dapa
2'-Deo
XIAP
53 kD
Caspase3
34 kD
Caspase8
57 kD
Caspase9
49 kD
BCL2
26 kD
BAX
20 kD
α-tubulin
55 kD

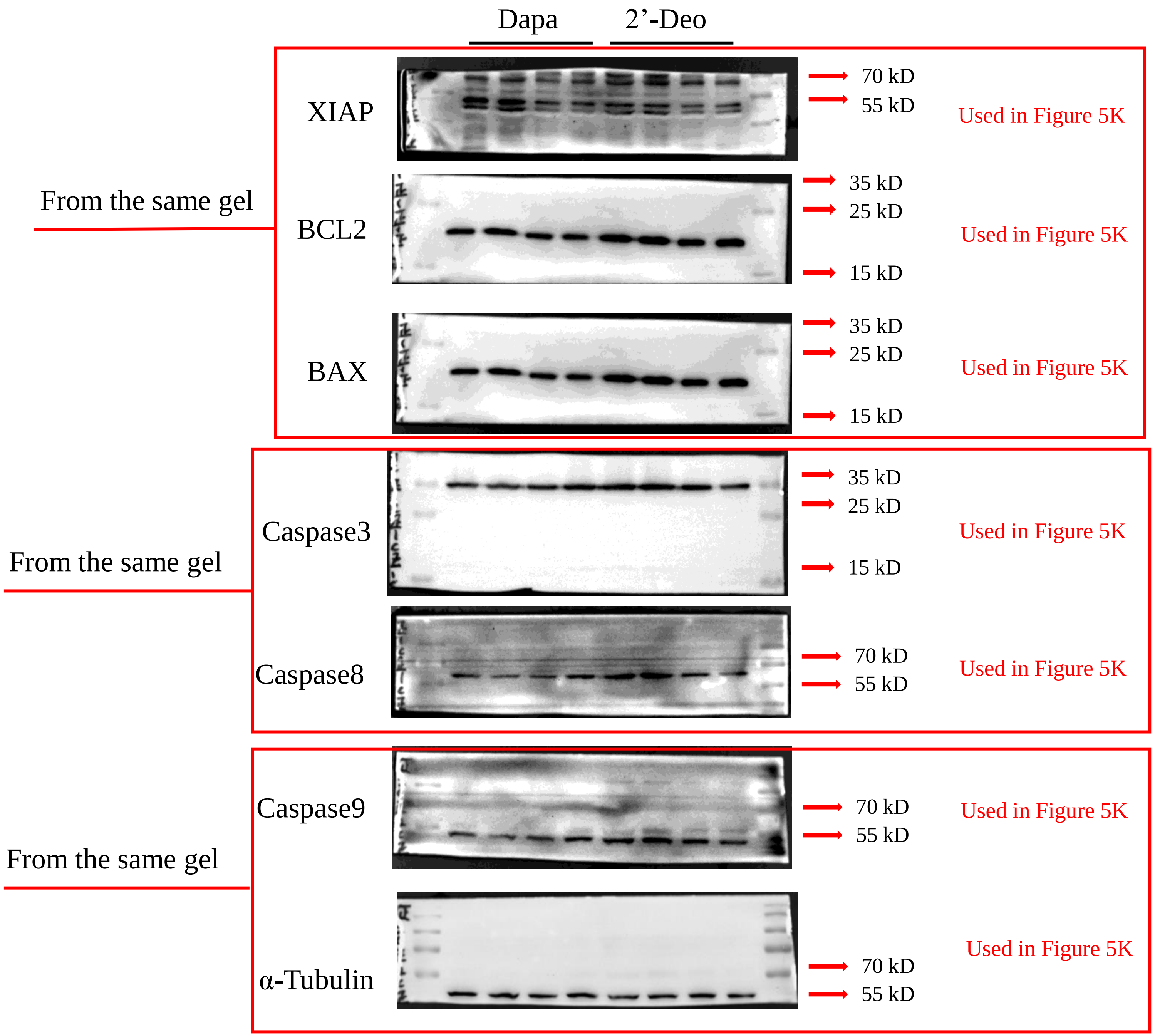
Dapa
2'-Deo
From the same gel
XIAP
70 kD
55 kD
Used in Figure 5K
BCL2
35 kD
25 kD
15 kD
Used in Figure 5K
BAX
35 kD
25 kD
15 kD
Used in Figure 5K
From the same gel
Caspase3
35 kD
25 kD
15 kD
Used in Figure 5K
Caspase8
70 kD
55 kD
Used in Figure 5K
From the same gel
Caspase9
70 kD
55 kD
Used in Figure 5K
α-Tubulin
70 kD
55 kD
Used in Figure 5K
