# Supplementary material for: Dapagliflozin ameliorates diabetes-induced spermatogenic dysfunction by modulating the adenosine metabolism along the gut microbiota-testis axis
Source: Sci Rep. 2024 Jan 5;14:641. doi: 10.1038/s41598-024-51224-2 (PMC10770392; doi:10.1038/s41598-024-51224-2)
Supplement: Supplementary file 2 — Supplementary Figures. [file 41598_2024_51224_MOESM2_ESM.docx]

**Dapagliflozin ameliorates diabetes-induced spermatogenic dysfunction by modulating the adenosine metabolism along the gut microbiota-testis axis**

Zirun Jin^a, b†^, Yalei Cao^a†^, Qi Wen^c, d, e, f†^, Haitao Zhang^a, c^, Zhuofan Fang^c, d, e, f^, Qiancheng Zhao^a^, Yu Xi^a^, Zhichao Luo^a^, Hui Jiang ^b^*, Zhe Zhang^a, c^*, and Jing Hang^c, d, e, f^ *

**Supplementary Data S1: Non-targeted metabolome of the gut, plasma, and testis samples of mice. Non-targeted metabolome profiling of the intestine, plasma, and testis samples was listed.**

**Supplemental figure S1–S11 and Figure legends**

**Supplemental figure S1. Dapagliflozin treatment had no effect on the sperm motility parameters of *db/db* diabetic mice.**

**Supplemental figure S2. Alterations in the taxonomic composition of microbial communities at the phylum and genus levels.**

**Supplemental figure S3. Alterations in the taxonomic composition of microbial communities at the species level.**

**Supplemental figure S4. The k-means clustering of microbial, plasmatic and testicular metabolites.**

**Supplemental figure S5. Alterations of the metabolite caused by dapagliflozin administration in *db/db* diabetic mice.**

**Supplemental figure S6.** **Molecular function analysis of all the regulated proteins.**

**Supplemental figure S7. The individual protein expression of XIAP, Caspase 3, Caspase 8, Caspase 9, BCL2 and BAX in testis tissues of Con, db and Dapa groups.**

**Supplemental figure S8. Alterations of oxidative stress related-metabolites in testis tissues.**

**Supplemental figure S9. Multi-omics factor analysis of intestinal metagenome, metabolome in intestine, plasma, testis samples and testis proteome.**

**Supplemental figure S10. Establishment of the diabetic GC-2 spd cells by palmitic acid (PA) treatment.**

**Supplemental figure S11. The individual protein expression of XIAP, Caspase 3, Caspase 8, Caspase 9, BCL2 and BAX in GC-2 cells of Vehicle, PA and Dapa groups.**


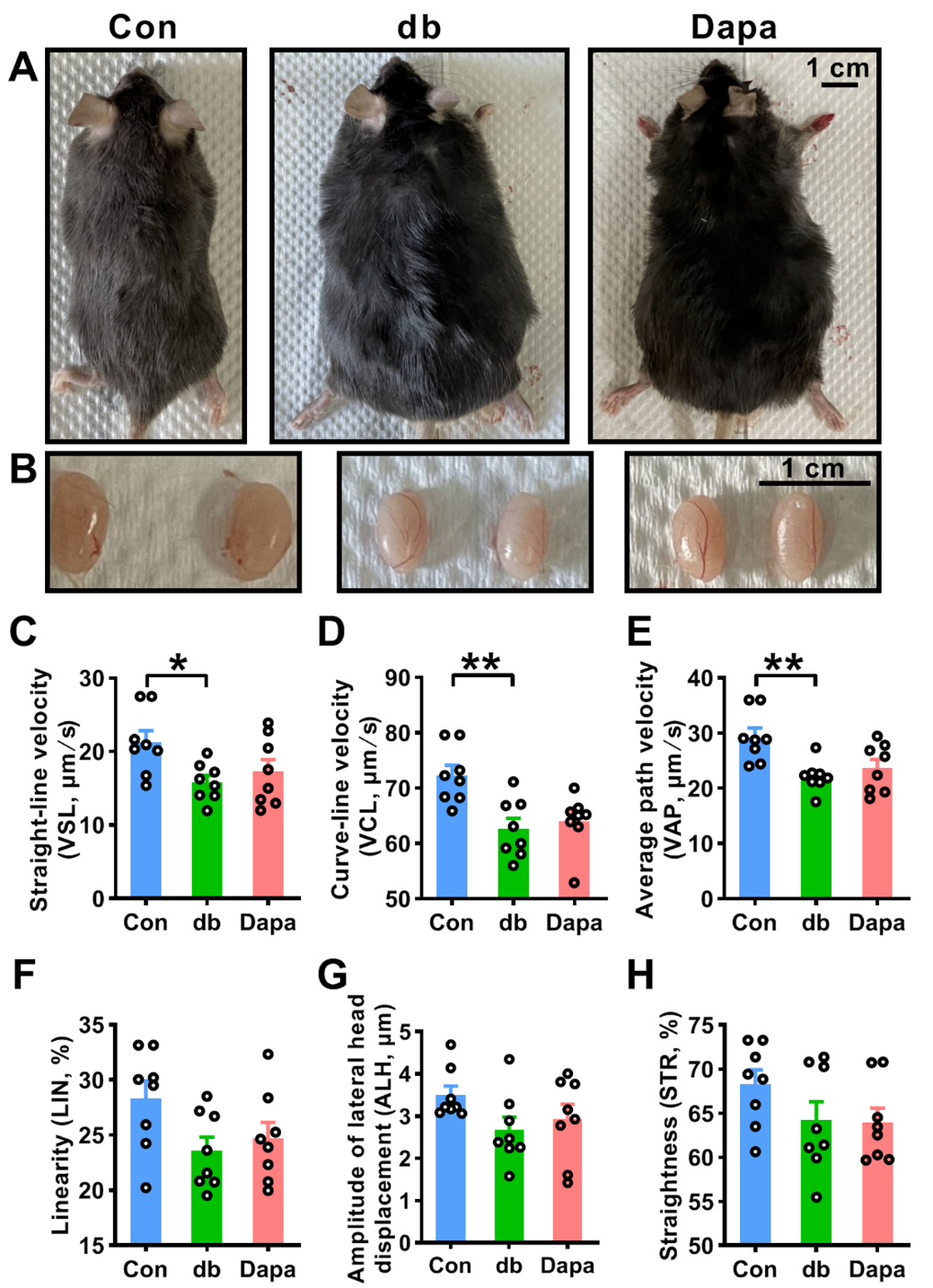


**Supplemental figure S1. Dapagliflozin treatment had no effect on the sperm motility parameters of *db/db* diabetic mice.** (**A–B**) Representative images of mice (A) and testis (B). (**C–H**) Other parameters of sperm motility such as straight-line velocity (VSL) (C), curve-line velocity (VCL) (D), average path velocity (VAP) (E), linearity (LIN) (F), amplitude of lateral head displacement (ALH) (G), and straightness (STR) (H). n = 8 mice per group. P values were determined by two-tailed unpaired Student's *t* test. *P < 0.05; **P < 0.01.


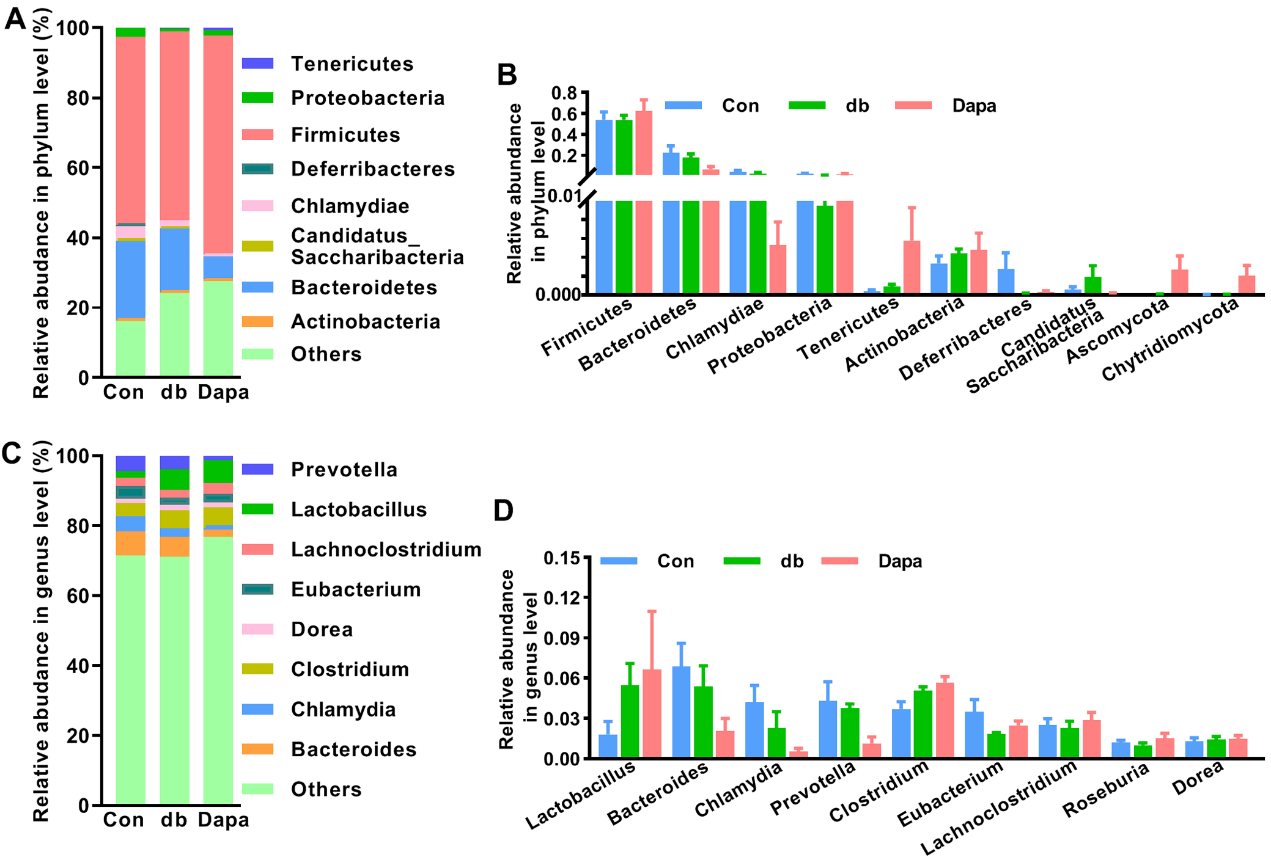
**Supplemental figure S2. Alterations in the taxonomic composition of microbial communities at the phylum and genus levels.** (**A–B**) The top nine maximum abundances (A) and changes in the abundance of bacteria (B) at the phylum level. (**C–D**) The top ten maximum abundance (C) and changes in abundance of bacteria (D) at the genus level.


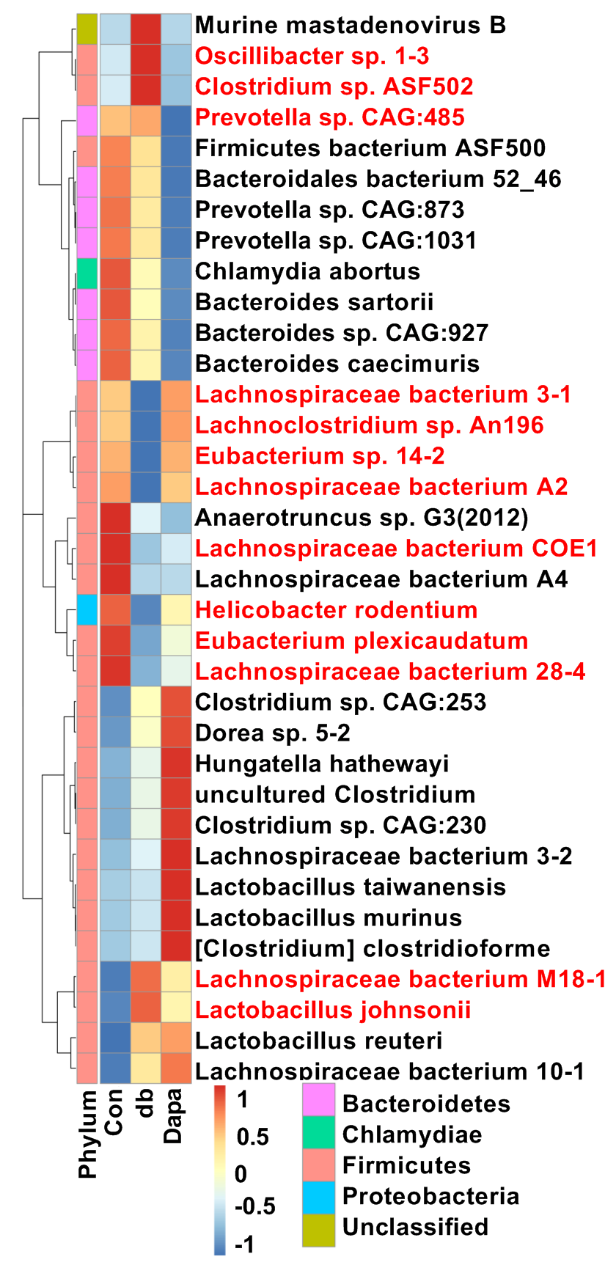
**Supplemental figure S3. Alterations in the taxonomic composition of microbial communities at the species level.** Average heatmap of the hierarchical clustering analysis for three groups. Abscissas represent different groups, and ordinates represent different species. n = 6 mice per group. Red font indicates the species that were upregulated in the db group and downregulated in the Dapa group or downregulated in the db group and upregulated in the Dapa group.


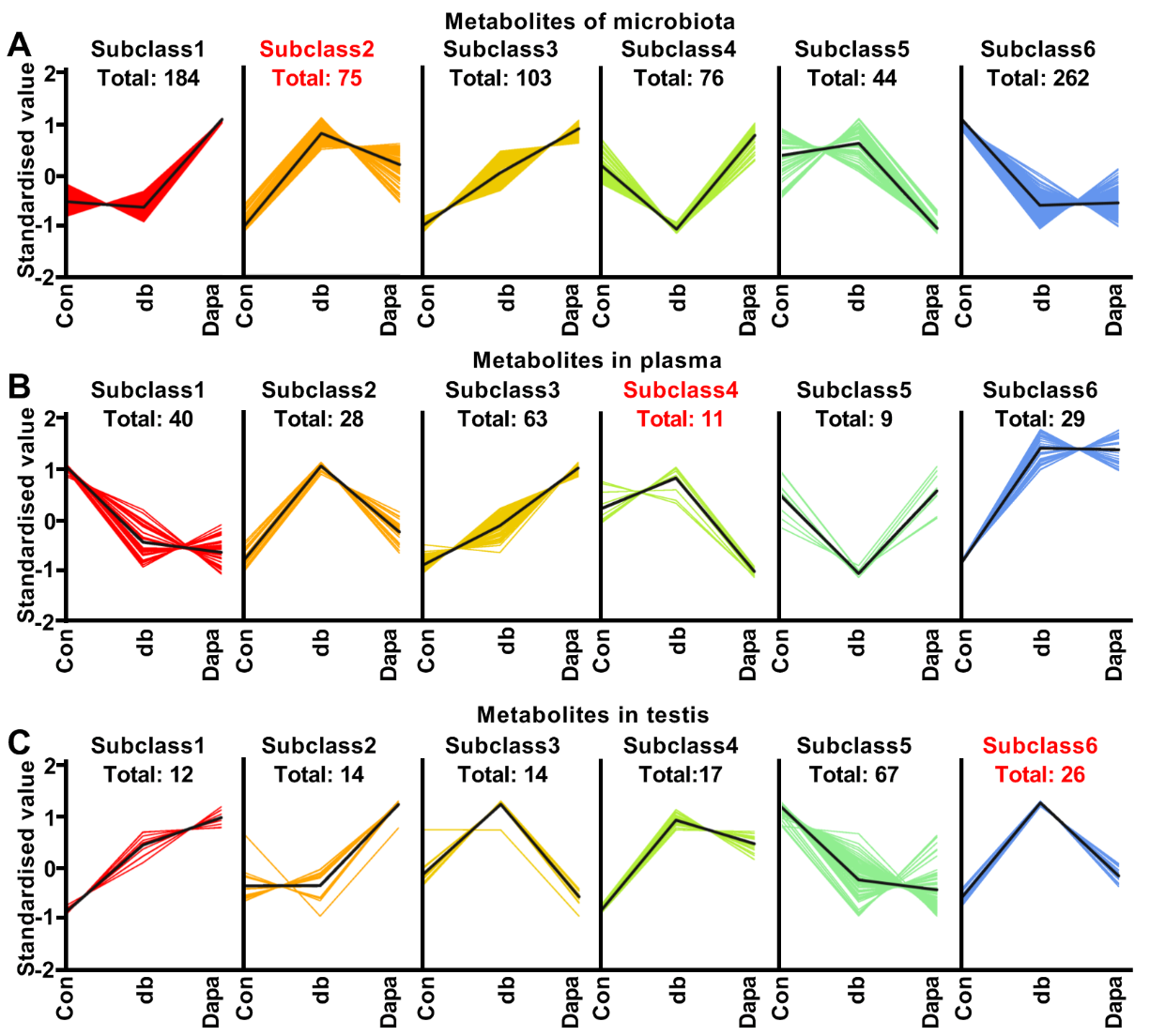
**Supplemental figure S4. The k-means clustering of microbial, plasmatic and testicular metabolites.** (**A–C**) The metabolites of the microbiota (A), plasma (B) and testis (C) of the three groups. n = 6 mice per group. The subclasses that include adenosine of microbiota, cAMP in plasma, and 2’-deoxyinosine in testis tissues are indicated in red.


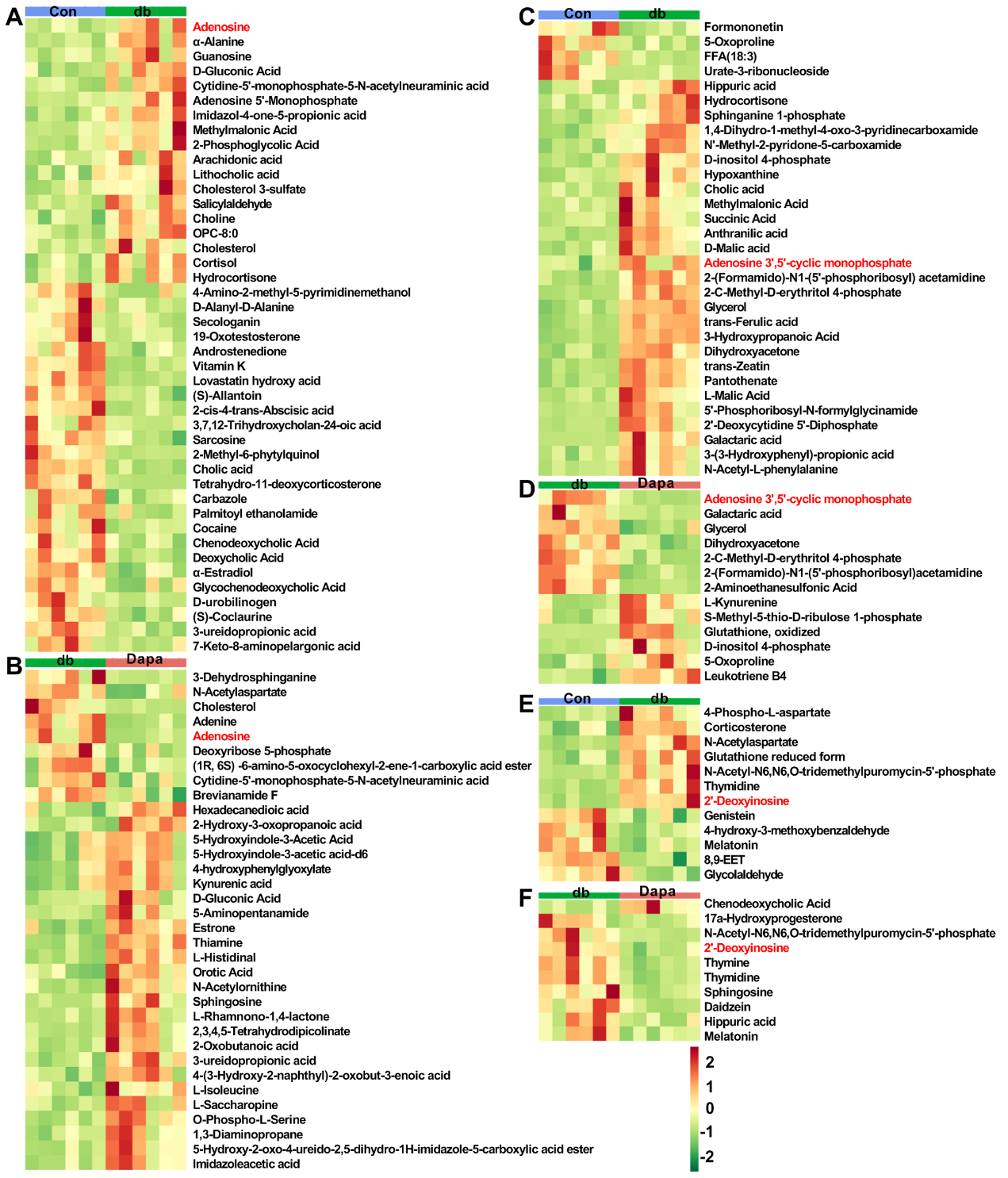
**Supplemental figure S5. Alterations of the metabolite caused by dapagliflozin administration in *db/db* diabetic mice.** Heatmaps of the hierarchical clustering analysis between the Con and db groups or between the db and Dapa groups. Metabolites of microbiota (A–B), plasma (C–D) and testis (E–­F). Abscissas represent different groups, and ordinates represent different metabolites. n = 6 mice per group. Red font indicates that the metabolites belong to the same pathway among the three tissues.


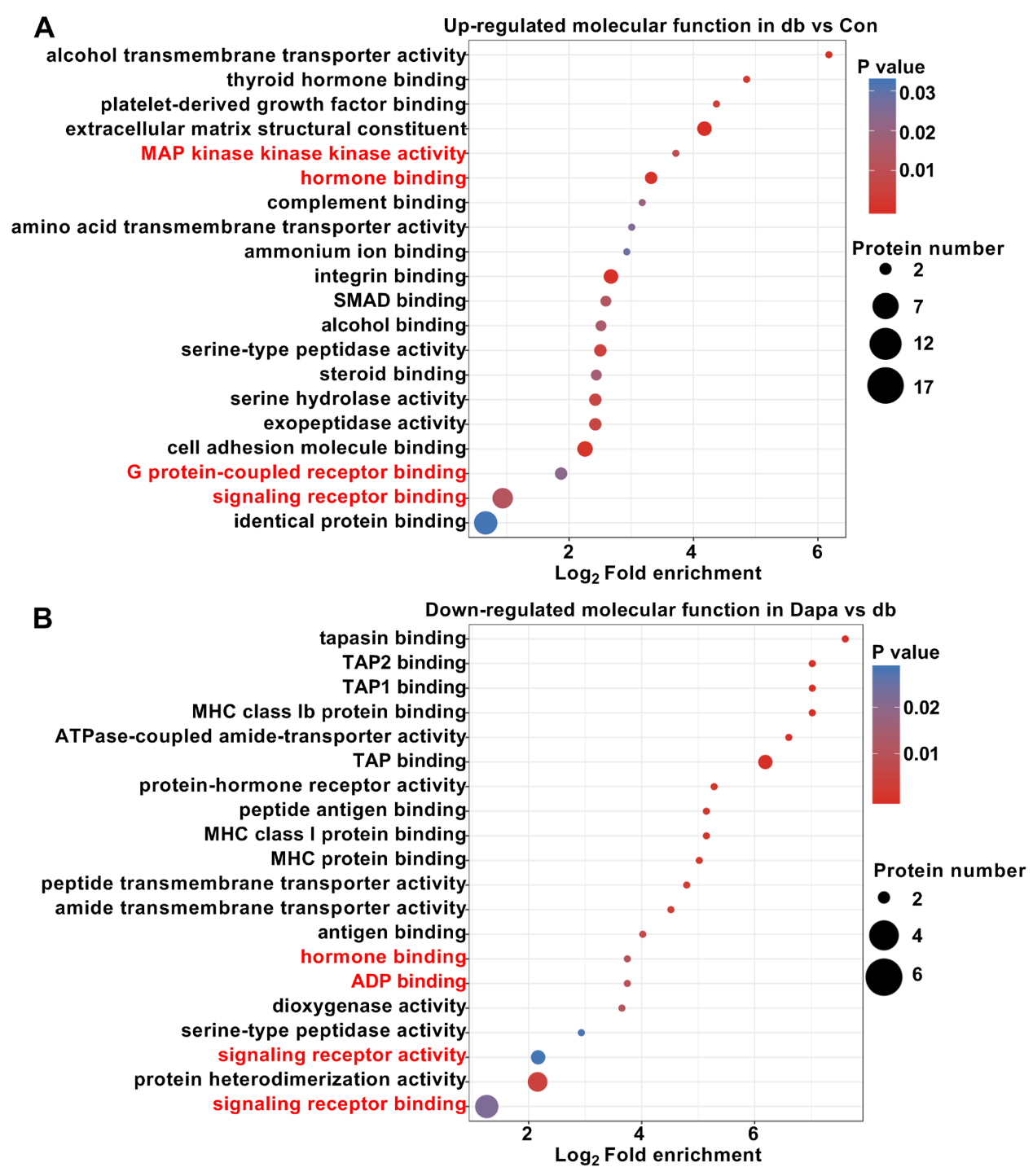
**Supplemental figure S6.** **Molecular function analysis of all the regulated proteins.** Molecular function (MF) enrichment clustering of differentially expressed proteins that were regulated in the db group or Dapa group. n = 6 mice per group.


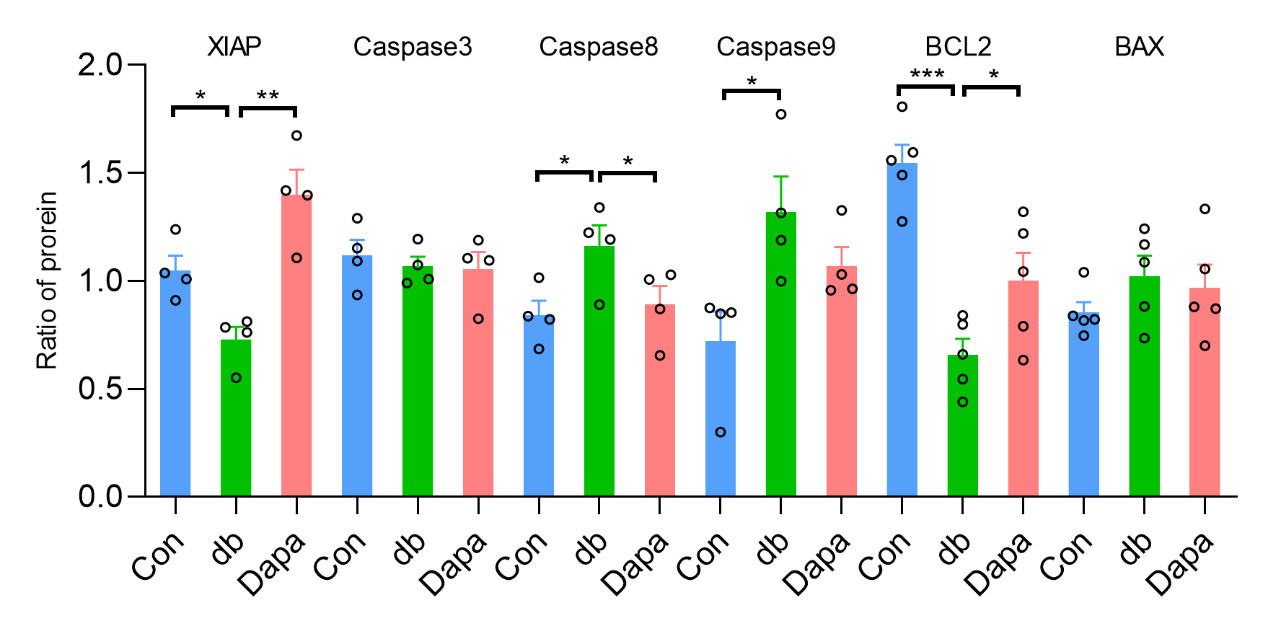
**Supplemental figure S7. The individual protein expression of XIAP, Caspase 3, Caspase 8, Caspase 9, BCL2 and BAX in testis tissues of Con, db and Dapa groups.** n = 4–5 mice per group. P values were determined by One-way ANOVA with Sidak's post-hoc test, *P < 0.05; **P < 0.01; ***P < 0.001, compared with Con or Dapa group.


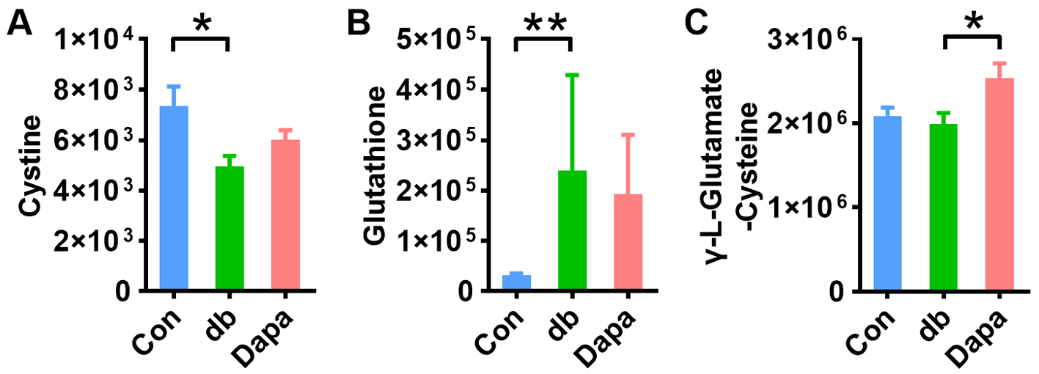
**Supplemental figure S8. Alterations of oxidative stress related-metabolites in testis tissues.** (**A–C**) The intensity of cysteine (A), glutathione (B), and γ-L-Glutamate-Cysteine (C) of con, db and Dapa groups, n = 6 mice per group. P values were determined by two-tailed unpaired Student's *t* test. *P < 0.05; **P < 0.01.


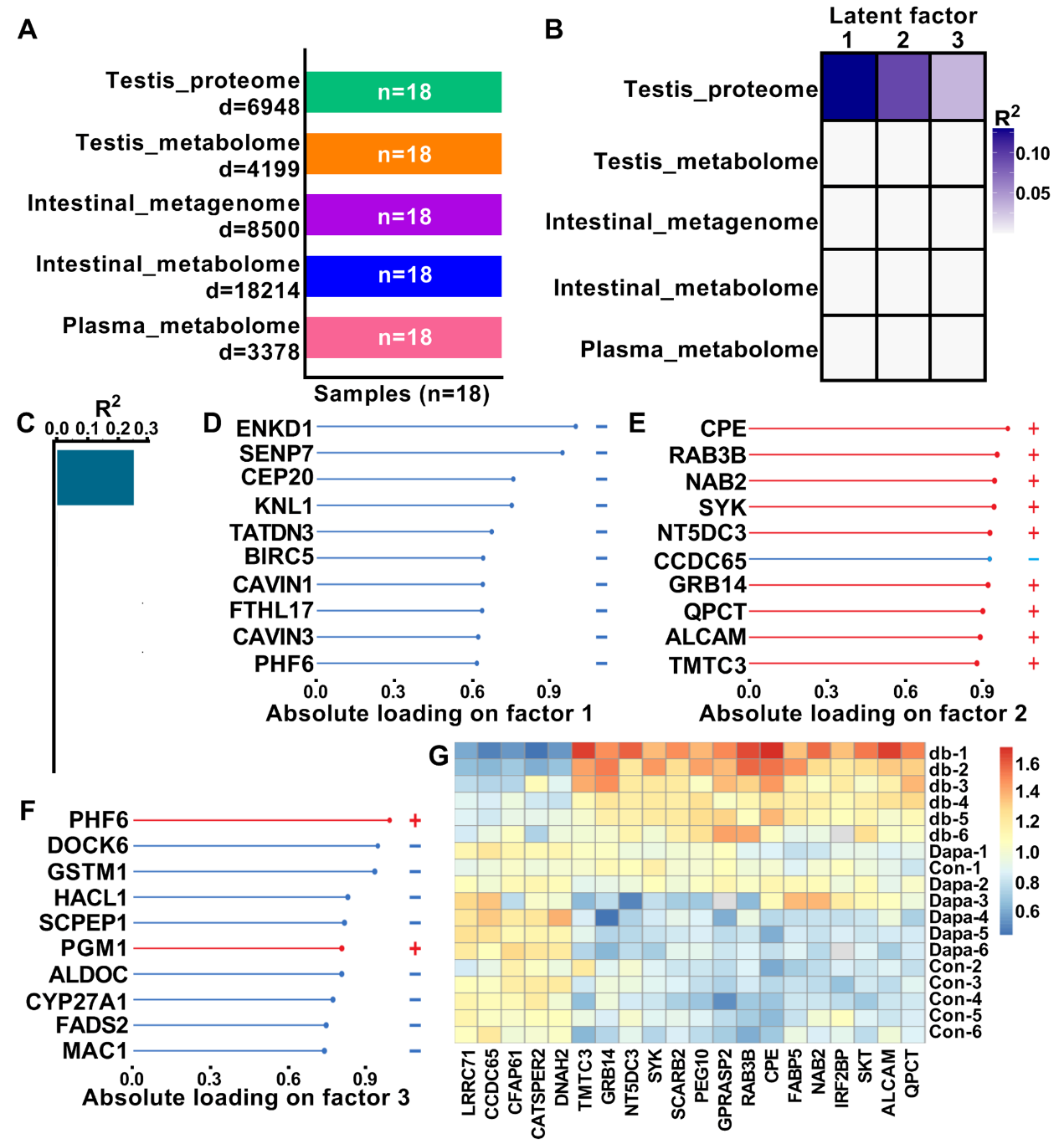
**Supplemental figure S9. Multi-omics factor analysis of intestinal metagenome, metabolome in intestine, plasma, testis samples and testis proteome.** (A) Study overview of MOFA analysis. Data modalities are shown in rows (d = number of features) and samples (n) in columns. (B) Proportion of total variance explained (R^2^) by individual factors for each assay. (C) Cumulative proportion of total variance explained. (**D–F**) Absolute loadings of the top features of factors 1 to 3 in the proteomic data. (G) Heatmap of the top 20 proteins of factor 2 from proteome array.


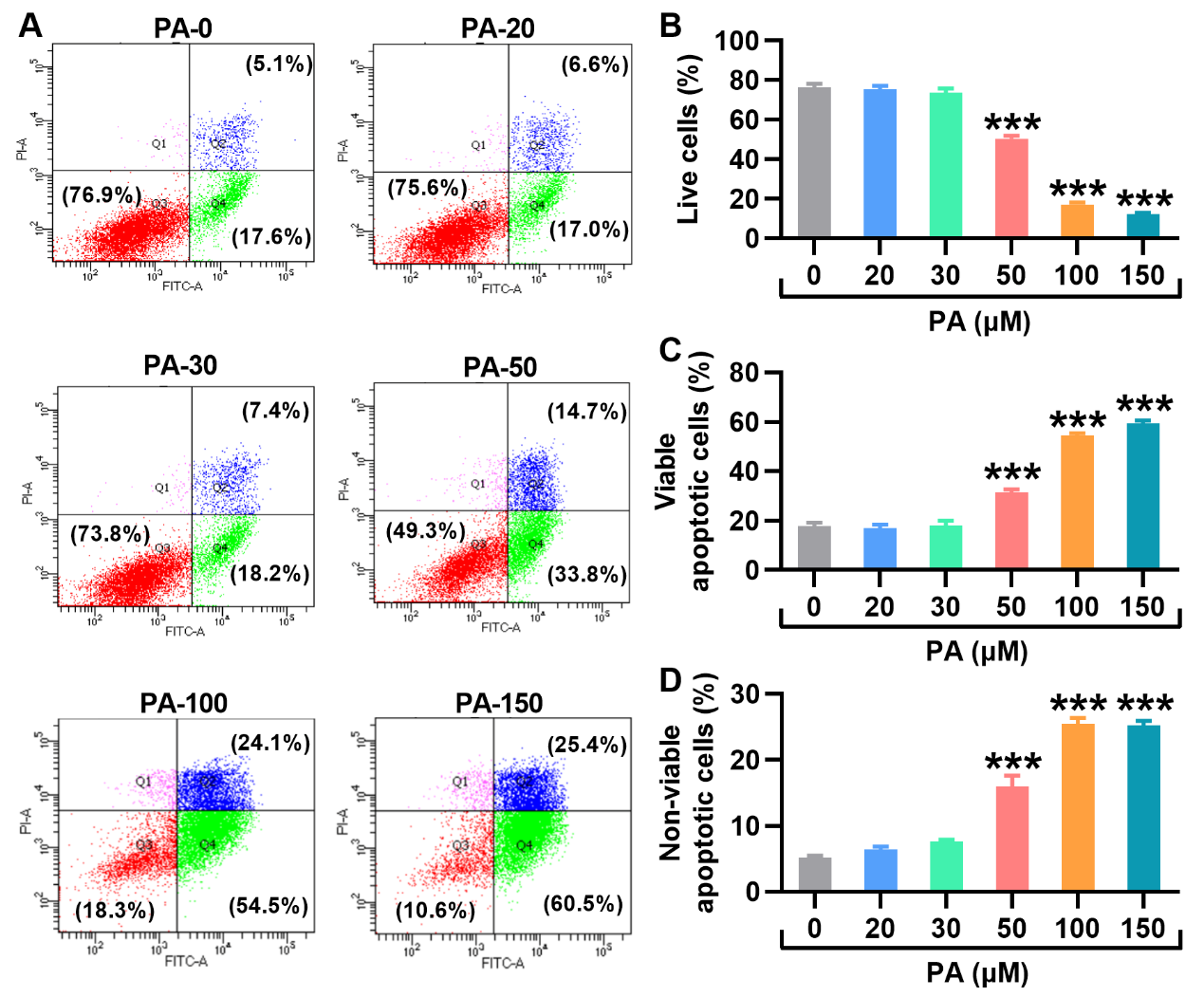
**Supplemental figure S10. Establishment of the diabetic GC-2 spd cells by palmitic acid (PA) treatment.** (**A–D**) Representative (A) and statistical analysis (B–D) of flow cytometry data after the treatment of different concentration of PA in GC-2 cells. n = 4 per group. P values were determined by two-tailed unpaired Student's *t* test. ***P < 0.001.


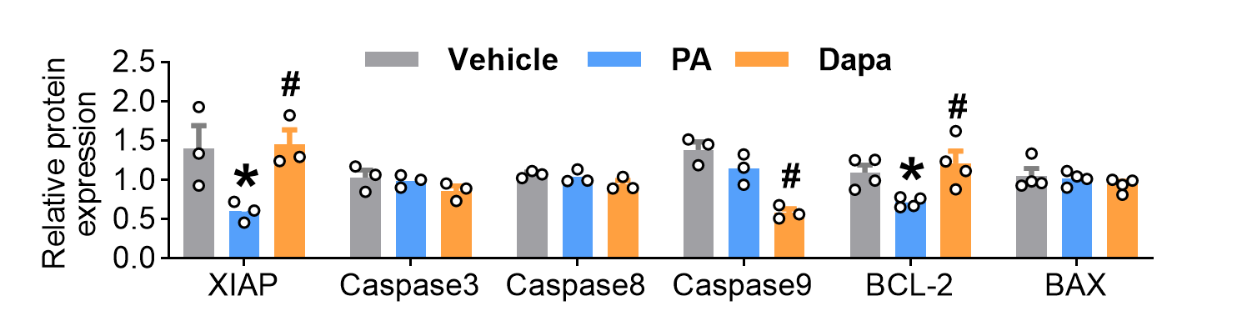


**Supplemental figure S11. The individual protein expression of XIAP, Caspase 3, Caspase 8, Caspase 9, BCL2 and BAX in GC-2 cells of Vehicle, PA and Dapa groups.** n = 3–4 per group. P values were determined by One-way ANOVA with Sidak's post-hoc test, *, #, P < 0.05, compared with Vehicle or Dapa group.
